# Questionnaires vs Interviews for the Assessment of Global Functional Outcomes After Traumatic Brain Injury

**DOI:** 10.1001/jamanetworkopen.2021.34121

**Published:** 2021-11-11

**Authors:** Lindsay Horton, Jonathan Rhodes, David K. Menon, Andrew I. R. Maas, Lindsay Wilson

**Affiliations:** 1 Division of Psychology, University of Stirling, Stirling, United Kingdom; 2 Department of Anaesthesia, University of Edinburgh, Western General, Edinburgh, United Kingdom; 3 Division of Anaesthesia, University of Cambridge, Addenbrooke’s Hospital, Cambridge, United Kingdom; 4 Department of Neurosurgery, Antwerp University Hospital and University of Antwerp, Edegem, Belgium

## Abstract

**Importance:**

An interview is considered the gold standard method of assessing global functional outcomes in clinical trials among patients with acute traumatic brain injury (TBI). However, several multicenter clinical trials have used questionnaires completed by a patient or caregiver to assess the primary end point.

**Objective:**

To examine agreement between interview and questionnaire formats for assessing TBI outcomes and to consider whether an interview has advantages.

**Design, Setting, and Participants:**

This cohort study used data from patients enrolled in the Collaborative European NeuroTrauma Effectiveness Research in TBI (CENTER-TBI) project from December 2014 to December 2017. Data were analyzed from December 2020 to April 2021. Included patients were aged 16 years or older with TBI and a clinical indication for computed tomography imaging. Outcome assessments were completed using both an interview and a questionnaire at follow-up 3 and 6 months after injury.

**Exposures:**

Traumatic brain injury of all severities.

**Main Outcomes and Measures:**

Ratings on the Glasgow Outcome Scale–Extended (GOSE) administered as a structured interview rated by an investigator and as a questionnaire completed by patients or caregivers and scored centrally were compared, and the strength of agreement was evaluated using weighted κ statistics. Secondary outcomes included comparison of different sections of the GOSE assessments and the association of GOSE ratings with baseline factors and patient-reported mental health, health-related quality of life, and TBI symptoms.

**Results:**

Among the 3691 eligible individuals in the CENTER-TBI study, both GOSE assessment formats (interview and questionnaire) were completed by 994 individuals (26.9%) at 3 months after TBI (654 [65.8%] male; median age, 53 years [IQR, 33-66 years]) and 628 (17.0%) at 6 months (409 [65.1%] male; median age, 51 years [IQR, 31-64 years]). Outcomes of the 2 assessment methods agreed well at both 3 months (weighted κ, 0.77; 95% CI, 0.73-0.80) and 6 months (weighted κ, 0.82; 95% CI, 0.78-0.86). Furthermore, item-level agreement between the 2 methods was good for sections regarding independence in everyday activities (κ, 0.70-0.79 across both time points) and moderate for sections regarding subjective aspects of functioning such as relationships and symptoms (κ, 0.41-0.51 across both time points). Compared with questionnaires, interviews recorded more problems with work (294 [30.5%] vs 233 [24.2%] at 3 months and 161 [26.8%] vs 136 [22.7%] at 6 months), fewer limitations in social and leisure activities (330 [33.8%] vs 431 [44.1%] at 3 months and 179 [29.7%] vs 219 [36.4%] at 6 months), and more symptoms (524 [53.6%] vs 324 [33.1%] at 3 months and 291 [48.4%] vs 179 [29.8%] at 6 months). Interviewers sometimes assigned an overall rating based on judgment rather than interview scoring rules, particularly for patients with potentially unfavorable TBI outcomes. However, for both formats, correlations with baseline factors (ρ, −0.13 to 0.42) and patient-reported outcomes (ρ, 0.29 to 0.65) were similar in strength.

**Conclusions and Relevance:**

In this cohort study, GOSE ratings obtained by questionnaire and interview methods were in good agreement. The similarity of associations of the ratings obtained by both GOSE methods with baseline factors and other TBI outcome measures suggests that despite some apparent differences, the core information collected by both interviews and questionnaires was similar. The findings support the use of questionnaires in studies in which this form of contact may offer substantial practical advantages compared with interviews.

## Key Points

**Question:**

Does interviewing have added value compared with questionnaires in the assessment of functional outcomes in clinical research on traumatic brain injury (TBI)?

**Findings:**

In this cohort study of 3691 individuals with TBI, there was good agreement between interview and questionnaire methods of assessing TBI outcomes, and correlations with baseline factors and patient-reported outcomes were similar between the methods. The core outcome information from interviews and questionnaires was found to be substantially similar.

**Meaning:**

These findings suggest that in appropriate settings, questionnaires can be used to assess global functional outcomes after TBI and may offer substantial practical advantages compared with interviews.

## Teaser

This cohort study compares global functional outcome ratings of the Glasgow Outcome Scale–Extended administered as a structured interview vs a questionnaire to patients with traumatic brain injury.

## Introduction

A popular way of assessing outcomes for clinical trials in acute traumatic brain injury (TBI) is through a clinician rating scale, particularly the Glasgow Outcome Scale (GOS) or Glasgow Outcome Scale–Extended (GOSE).^1^ A structured interview has become a standard method for obtaining ratings on the GOSE and is a core recommended outcome in the Common Data Elements for TBI.^2^

A questionnaire version of the GOSE, completed by the patient or a caregiver, has been used as an end point in several multicenter trials of acute TBI.^3,4,5,6^ The questionnaire format avoids investigator bias in studies such as surgical trials where masking is impractical. Questionnaires offer pragmatic advantages in overall costs and can make large-scale clinical trials of TBI feasible if industry sponsorship is lacking.^7^ Although there have been concerns about low follow-up rates,^8^ 6-month GOSE outcomes were obtained for 97% of patients enrolled in the Eurotherm3235 Trial.^3^ In practice, studies have typically followed up nonresponders by telephone interview or another type of contact that can be organized centrally. These studies thus ultimately combined ratings derived from questionnaires and interviews in their primary end point.

Work^9^ to date comparing GOSE interviews and questionnaires has been small in scale and did not indicate whether there were differences in the information collected or whether an interview offered added value compared with a questionnaire. Interviewing may be expected to be superior because it allows for flexible questioning in borderline cases, the reliability of respondents can be evaluated, and when inconsistencies arise, a judgment can be made concerning the overall rating. Areas likely to need judgment include the influence of preexisting disability or extracranial injury.^10^ If interviews are superior, the ratings should have better validity, for example, by identifying dependency more precisely or by discounting preexisting disability. Interview ratings should be more correlated with measures of injury severity than questionnaires completed by patients and caregivers. The latter, in contrast, might be expected to be more subjective and more correlated with patient-reported outcomes.

The Collaborative European NeuroTrauma Effectiveness Research in TBI (CENTER-TBI) project^11^ used a flexible data-collection approach to maximize follow-up. The GOSE was administered as either an interview or a questionnaire. To allow comparison of methods, the study design encouraged CENTER-TBI investigators to collect both versions of the GOSE when possible. In addition, patient-reported outcomes were used to assess health-related quality of life, mental health, and TBI symptoms. The information that was available to investigators at the time of the interview could include completed questionnaires. Thus, the comparison made in the current study concerns whether interviewing added value to the GOSE assessment and increased validity. We compared agreement of the assessments in 3 areas: (1) overall ratings, (2) individual sections of the GOSE, and (3) correlations with baseline factors and patient-reported outcomes. We also studied the use of judgment by interviewers in assigning an overall rating.

## Methods

This cohort study used data from patients enrolled in the CENTER-TBI project from December 2014 to December 2017. Data were analyzed from December 2020 to April 2021. Ethical approval was obtained for each project site according to national and local procedures. A detailed ethics statement is given on the project website.^12^ This study followed the Strengthening the Reporting of Observational Studies in Epidemiology (STROBE) reporting guideline.

### Participants

The CENTER-TBI project included 4509 patients from 65 sites across 18 countries.^13^ Inclusion criteria were a diagnosis of TBI and clinical indication for a computed tomography scan, being seen in a hospital within 24 hours of the injury, and availability of informed consent (written consent was obtained at the earliest opportunity, but some patients may have been enrolled initially with oral consent). Patients were excluded if they had a severe preexisting neurological disorder that would confound outcome assessments. Additional inclusion criteria for the current analyses were an age of 16 years or older, survival at 6 months after injury, a complete and scorable GOSE interview and questionnaire for the participant at 3 months or 6 months after injury, and completion of the GOSE interview and questionnaire within 3 weeks of one another.

### Measures

Demographic information was recorded at the time of recruitment along with information on the cause of injury and preexisting systemic disease based on the American Society of Anesthesiologists Physical Status Classification System.^14^ Injury severity was assessed using early computed tomography imaging,^15^ the Abbreviated Injury Scale score (scores range from 1 to 6, with higher scores indicating more severe injury) and Injury Severity Score (scores range from 1 to 75, with higher scores indicating more severe injury),^16^ and a baseline Glasgow Coma Scale score (scores range from 3 to 15, with higher scores indicating less severe injury).^17,18^

#### Global Outcome

The GOSE interview^19^ was administered at each site either in person or by telephone. Investigators attended training and were provided a study manual including advice on supplementary questions for borderline situations, problem cases, and scoring.^10^ Interviewers were instructed to include disability associated with all aspects of the injury, including extracranial injury, in the rating. The weighted κ statistic (κ_w_) for test-retest agreement is 0.92.^20^

The GOSE questionnaire^9^ consists of 14 questions in 7 sections that parallel the interview (eAppendix in the Supplement 1). Questions are designed to be appropriate for an adult patient or caregiver. The Flesch readability score for the text is 72, and the Flesch-Kincaid Grade Level is 6.5. The response choices include the option to indicate that limitations are present but are not attributable to head injury; responses using this option are not included in the scoring. Because it is not practical to assess responsiveness using a questionnaire format, the categories of “lower severe disability” and “vegetative state” are collapsed. The κ_w_ for test-retest agreement is 0.98.^9^

#### Health-Related Quality of Life

The 36-Item Short Form Health Survey, version 2 (SF-36v2)^21^ is a patient-reported outcome that has been used for many health conditions. The instrument has 8 subscales and 2 summary scores, the Mental Component Summary and the Physical Component Summary. Scores are transformed to T-scores (mean [SD], 50 [10]), with higher scores indicating better outcomes.

The Quality of Life after Brain Injury Scale^22^ is a TBI-specific measure of health-related quality of life comprising 37 items in 6 domains relevant for brain injury. Scores range from 0 to 100, with higher scores indicating better health-related quality of life.

#### Mental Health

The Patient Health Questionnaire–9 is a self-report instrument of 9 items assessing depression severity.^23^ Scores range from 0 to 27, with higher scores indicating greater depression. The Generalized Anxiety Disorder–7 is a 7-item self-report instrument for the severity of anxiety symptoms.^24^ Scores range from 0 to 21, with higher scores indicating greater anxiety.

#### TBI Symptoms

The Rivermead Post Concussion Symptoms Questionnaire is a self-report instrument consisting of 16 symptoms typical after mild or moderate TBI.^25^ Scores range from 0 to 64, with higher scores indicating a greater burden of symptoms. Comparisons with this questionnaire were restricted to patients with a baseline Glasgow Coma Scale score of 9 to 15 (ie, mild or moderate injury), consistent with the context of use of this instrument. When translations were not available from the publishers, all instruments used were translated into local languages using a process of linguistic validation.^26^

### Data Collection Procedure

Patients were enrolled from December 2014 to December 2017. Follow-up was scheduled at 3 and 6 months. The 3-month assessment was conducted either in person or by a postal questionnaire and telephone interview. The 6-month assessment was planned as an in-person meeting that included the GOSE interview; questionnaires could be completed at the time of follow-up or returned by post. To allow sites the flexibility to maximize follow-up, the use of both interviews and questionnaires was not mandated, but investigators were encouraged to collect both versions if possible.

### Statistical Analysis

Data were downloaded on November 22, 2019, from the Neurobot database, version 2.1 (International Neuroinformatics Coordinating Facility). Analyses were conducted from December 2020 to April 2021 using IBM SPSS, version 25 (IBM). Demographic and clinical characteristics were described using frequencies and percentages for categorical variables and medians and IQRs for continuous data.

#### Agreement Between Instruments

Preinjury and postinjury items in each of the 7 subsections of the GOSE were used to code whether a problem or limitation was recorded that had not been present before the injury. The strength of agreement for these 2 × 2 comparisons was evaluated using the κ statistic^27^ (≤0.20, poor; 0.21-0.40, fair; 0.41-0.60, moderate; 0.61-0.80, good; and 0.81-1.00, very good).^28^ Differences in limitations recorded in each section were evaluated by the McNemar test. Agreement between overall ratings was assessed using κ_w_; quadratic weights penalize extreme disagreements between ratings more heavily than slight disagreements.^29^ The Wilcoxon signed rank test was used to test for differences between GOSE scores from the 2 formats, with *r* as the measure of effect size. We also compared the questionnaire and interview formats when ratings were dichotomized between upper severe disability and lower moderate disability, a common cut point for unfavorable vs favorable outcomes.

To provide an indication of the use of personal judgment in assigning overall ratings for the interviews, we identified departure from interview scoring rules. We scored the interviews centrally according to the standard procedure for the assessment and calculated the difference between interviewer ratings and central scoring. Variation in these differences across GOSE outcome categories (assigned by central scoring) was assessed using χ^2^ tests.

#### Comparative Validity

Spearman correlations were calculated between the GOSE and baseline factors typically included in prognostic models (ie, age, Glasgow Coma Scale score, pupillary reactivity, Injury Severity Score, Abbreviated Injury Scale score, and extracranial injury) and patient-reported outcome measures (ie, measures of health-related quality of life, psychological status, and TBI symptoms). A percentile bootstrap method^30^ was used to ascertain whether pairs of correlations were significantly different. Because multiple comparisons were made, statistical tests were considered significant only if 2-tailed *P* < .01.

## Results

Of 3691 individuals aged 16 years or older who were alive and eligible for follow-up 6 months after injury (eFigure in the Supplement 1), 994 (26.9%) completed both assessments at 3 months (654 [65.8%] male; median age, 53 years [IQR, 33-66 years]) and 628 (17.0%) completed both assessments at 6 months (409 [65.1%] male; median age, 51 years [IQR, 31-64 years]). The demographic and clinical characteristics of the individuals are summarized in Table 1.

**Table 1.** Demographic and Clinical Characteristics of Respondents

Characteristic	Respondents, No. (%)^a^		
	3-mo Follow-up (n = 994)	6-mo Follow-up (n = 628)	Total eligible (N = 3691)
Age, median (IQR), y	53 (33-66)	51 (31-64)	49 (31-64)
Sex			
Male	654 (65.8)	409 (65.1)	2487 (67.4)
Female	340 (34.2)	219 (34.9)	1204 (32.6)
Race and ethnicity			
Asian	12 (1.2)	7 (1.1)	56 (1.6)
Black	12 (1.2)	5 (0.8)	57 (1.6)
White	946 (97.5)	605 (98.1)	3419 (96.8)
Missing or unknown	24 (2.4)	11 (1.8)	159 (4.3)
Educational level			
Primary	90 (10.8)	65 (13.1)	459 (14.8)
Secondary	275 (32.9)	183(36.9)	1108 (35.7)
Training	202 (24.2)	103 (20.8)	646 (20.8)
College	268 (32.1)	145 (29.2)	888 (28.6)
Missing	159 (16.0)	132 (21.0)	590 (16.0)
Employment status before injury			
Working full or part time	476 (52.0)	310 (55.3)	1804 (53.7)
Not working	71 (7.8)	52 (9.3)	317 (9.4)
Retired	257 (28.1)	134 (23.9)	828 (24.7)
Student or homemaker	112 (12.2)	65 (11.6)	409 (12.2)
Missing	78 (7.8)	67 (10.7)	333 (9.0)
Marital status			
Partnered	524 (55.8)	303 (52.6)	1751 (51.5)
Previously partnered	132 (14.1)	77 (13.4)	520 (15.3)
Single or other	283 (30.1)	196 (34.0)	1126 (33.1)
Unknown	55 (5.5)	52 (8.3)	294 (8.0)
ASA preinjury physical health			
Healthy patient	592 (60.3)	367 (59.8)	2126 (58.9)
Mild systemic disease	312 (31.8)	196 (31.9)	1156 (32.0)
Severe systemic disease	77 (7.8)	51 (8.3)	325 (9.0)
Missing	13 (1.3)	14 (2.2)	84 (2.3)
Cause of injury			
Road traffic accident	407 (41.6)	256 (42.0)	1411 (39.2)
Fall	423 (43.3)	251 (41.2)	1617 (45.0)
Violence or assault	67 (6.9)	43 (7.1)	208 (5.8)
Other	81 (8.3)	59 (9.7)	361 (10.0)
Missing or unknown	16 (1.6)	19 (3.0)	94 (2.5)
Clinical care pathway			
Emergency department	179 (18.0)	129 (20.5)	774 (21.0)
Admitted to hospital	376 (37.8)	181 (28.8)	1324 (35.9)
Intensive care unit	439 (44.2)	318 (50.6)	1593 (43.2)
GCS score at baseline			
13-15	683 (72.0)	392 (66.3)	2624 (73.5)
9-12	93 (9.8)	65 (11.0)	285 (8.0)
3-8	172 (18.1)	134 (22.7)	659 (18.5)
Missing	46 (4.6)	37 (5.9)	123 (3.3)
CT imaging abnormal finding			
Present	525 (57.1)	338 (58.9)	1911 (56.7)
Absent	394 (42.9)	236 (41.1)	1462 (43.3)
Missing	75 (7.5)	54 (8.6)	318 (8.6)
Pupillary reactivity			
Both reactive	874 (94.8)	538 (92.9)	3238 (93.1)
1 Pupil unreactive	25 (2.7)	20 (3.5)	118 (3.4)
2 Pupils unreactive	23 (2.5)	21 (3.6)	121 (3.5)
Missing	72 (7.2)	49 (7.8)	214 (5.8)
Total ISS, median (IQR)	16 (9-26)	16 (8-29)	16 (9-26)
Head and neck AIS score^b^			
No injury or minor injury	167 (16.8)	112 (17.8)	655 (17.7)
Moderate injury	133 (13.4)	86 (13.7)	521 (14.1)
Serious injury	321 (32.3)	146 (23.2)	1128 (30.6)
Severe injury	165 (16.6)	119 (18.9)	637 (17.3)
Critical injury	206 (20.9)	165 (26.3)	750 (20.3)
Major extracranial injury^c^			
No injury or mild injury	643 (64.7)	424 (67.5)	2435 (66.0)
Severe injury	351 (35.3)	204 (32.5)	1256 (34.0)

Abbreviations: AIS, Abbreviated Injury Scale; ASA, American Society of Anesthesiologists; CT, computed tomography; GCS, Glasgow Coma Scale; ISS, Injury Severity Score.

^a^ Percentages for observed values exclude missing data from the calculations.

^b^ Combined AIS score for head, neck, and cervical regions.

^c^ Any non–head and neck AIS score of 3 or higher (serious injury).

### Agreement Between GOSE Scores

Details of GOSE outcome assessments are given in Table 2. Agreement between formats was good at 3 months (κ_w_, 0.77; 95% CI, 0.73-0.80) and very good at 6 months (κ_w_, 0.82, 95% CI, 0.78-0.86). There was exact agreement for 539 three-month ratings (53.8%) and 376 six-month ratings (61.6%) (eTable 1 in the Supplement 1). An additional 306 three-month ratings (30.8%) and 179 six-month ratings (28.5%) disagreed by only 1 GOSE category. Large discrepancies (ie, ≥3 GOSE categories) were uncommon and included 58 ratings (5.8%) in the 3-month sample and 25 (4.0%) in the 6-month sample. At the 3-month follow-up, the interview rating was greater than that of the questionnaire for 195 patients (19.6%) and less than that of the questionnaire for 264 (26.6%), whereas at the 6-month follow-up, the interview rating was greater for 86 patients (13.7%) and lesser for 155 patients (24.7%). Wilcoxon signed rank tests comparing formats indicated that median GOSE scores were not significantly different at 3 months’ follow-up (*Z*, −1.45; *r*, 0.05; *P* = .15), whereas the difference was significant at 6 months’ follow-up (*Z*, −3.88; *r*, −0.15, *P* < .001).

**Table 2.** Assessment Characteristics and Respondents

Characteristic	Respondents, No. (%)	
	3-mo Follow-up (n = 994)	6-mo Follow-up (n = 628)
Interval between assessments, d		
Same day	547 (55.0)	483 (76.9)
1-7	217 (21.8)	88 (14.0)
8-14	145 (14.6)	34 (5.4)
15-21	85 (8.6)	23 (3.7)
Order of assessments		
Interview ≥1 d before questionnaire	297 (29.9)	68 (10.8)
Interview and questionnaire on same day	547 (55.0)	483 (76.9)
Interview ≥1 d after questionnaire	150 (15.2)	77 (12.3)
GOSE interview respondent		
Patient alone	766 (78.2)	494 (81.3)
Relative, friend, or caregiver alone	134 (13.7)	61 (10.0)
Patient plus relative, friend, or caregiver	80 (8.2)	53 (8.7)
Missing	14 (1.4)	20 (3.2)
GOSE questionnaire respondent		
Patient alone	738 (74.4)	504 (80.4)
Relative, friend, or caregiver alone	97 (9.8)	59 (9.4)
Patient plus relative, friend, or caregiver	157 (15.8)	64 (10.2)
Missing	2 (0.2)	1 (0.2)

Abbreviation: GOSE, Glasgow Outcome Scale–Extended.

Additional comparisons were made to examine subgroups divided by preexisting functional limitations, concomitant extracranial injury, and level of education (eTable 2 in the Supplement 1). There was at least good agreement (ie, κ_w_, ≥0.70) between GOSE assessments for the subgroup comparisons; there was greater disagreement for patients with preexisting systemic illness than for those without.

Scores dichotomized between upper severe disability and lower moderate disability were in good agreement at 3 months (κ, 0.65; 95% CI, 0.59-0.71) and 6 months (κ, 0.75; 95% CI, 0.68-0.82). At 3 months, fewer outcomes were classified as unfavorable by the interview than by the questionnaire (30 [3.0%] vs 79 [7.9%]; *P* < .001), whereas at 6 months, this difference was no longer significant (14 [2.2%] vs 26 [4.1%]; *P* = .08) (eTable 3 in the Supplement 1).

A difference of 1 or more categories between the interviews scored centrally and the ratings assigned by interviewers was present in 257 three-month scores (26.2%) and 143 six-month scores (23.7%). There was a significant association between discrepancies and GOSE category at both 3 months (χ^2^, 226; *P* < .001) and 6 months (χ^2^, 213; *P* < .001) (eTable 4 in the Supplement 1). Interviewers tended to rate the patients classified by central scoring as having unfavorable outcomes as less disabled (particularly in GOSE category 4). Interviewers also tended to rate the patients at the upper end of the scale as more disabled (particularly GOSE category 8).

### Ratings for Sections of the GOSE

Levels of agreement in individual sections of the interview and questionnaire are shown in Table 3 (frequency counts are shown in eTable 5 in the Supplement 1). Sections regarding independence at home, shopping, and travel had good levels of agreement (κ, 0.70-0.79 across both time points), as did sections regarding work and participation in social and leisure activities (κ, 0.60-0.74 across both time points). Personal relationships and symptoms that interfered with daily life had moderate levels of agreement (κ, 0.41-0.51 across both time points).

**Table 3.** Agreement Between Individual Sections of the Glasgow Outcome Scale–Extended Interview and Questionnaire at the 3-Month and 6-Month Follow-up

Section	Responses, No.	Exact agreement, No. (%)	κ (95% CI)
3-mo Follow-up			
Assistance at home	979	891 (91.0)	0.70 (0.64-0.76)
Shopping	979	927 (94.7)	0.79 (0.75-0.85)
Travel	979	923 (94.3)	0.79 (0.74-0.85)
Work	964	847 (87.9)	0.70 (0.65-0.75)
Social and leisure	977	788 (80.7)	0.60 (0.55-0.65)
Relationships	973	866 (89.0)	0.50 (0.42-0.58)
Symptoms	978	702 (71.8)	0.45 (0.40-0.50)
6-mo Follow-up			
Assistance at home	603	561 (93.0)	0.73 (0.66-0.81)
Shopping	602	568 (94.4)	0.74 (0.65-0.82)
Travel	603	571 (94.7)	0.76 (0.68-0.84)
Work	600	541 (90.2)	0.74 (0.67-0.80)
Social and leisure	602	498 (82.7)	0.61 (0.55-0.68)
Relationships	603	525 (87.1)	0.51 (0.41-0.60)
Symptoms	601	425 (70.7)	0.41 (0.34-0.48)

The percentages of patients who had post-TBI limitations on each section of the GOSE are shown in the Figure. Limitations were most common for TBI-related symptoms (interview: 524 [53.6%] at 3 months and 291 [48.4%] at 6 months; questionnaire: 324 [33.1%] at 3 months and 179 [29.8%] at 6 months), social and leisure activities (interview: 330 [33.8%] at 3 months and 179 [29.7%] at 6 months; questionnaire: 431 [44.1%] at 3 months and 219 [36.4%] at 6 months), and work (interview: 294 [30.5%] at 3 months and 161 [26.8%] at 6 months; questionnaire: 233 [24.2%] at 3 months and 136 [22.7%] at 6 months) and least common for independence inside the home (interview: 179 [18.3%] at 3 months and 99 [16.4%] at 6 months; questionnaire: 173 [17.7%] at 3 months and 87 [14.4%] at 6 months), for shopping (interview: 158 [16.1%] at 3 months and 78 [13.0%] at 6 months; questionnaire: 146 [14.9%] at 3 months and 68 [11.3%] at 6 months), for travel (interview: 160 [16.3%] at 3 months and 72 [11.9%] at 6 months; questionnaire: 164 [16.8%] at 3 months and 78 [12.9%] at 6 months) and for relationships (interview: 117 [12.0%] at 3 months and 82 [13.6%] at 6 months; questionnaire: 128 [13.2%] at 3 months and 104 [17.2%] at 6 months). Interviewers recorded significantly more problems with work (294 [30.5%] vs 233 [24.2%]; *P* < .001) and fewer problems with social and leisure activities (330 [33.8%] vs 431 [44.1%]; *P* < .001) at 3 months than were reported on the questionnaires; these differences remained at 6 months but were reduced (work: 161 [26.8%] vs 136 [22.7%]; *P* = .002; social and leisure activities: 179 [29.7%] vs 219 [36.4%]; *P* < .001). At both time points, interviewers recorded the presence of symptoms that interfered with daily life more often than was reported on the questionnaires (524 [53.6%] vs 324 [33.1%] at 3 months [*P* < .001] and 291 [48.4%] vs 179 [29.8%] at 6 months [*P* < .001]).

**Figure.**
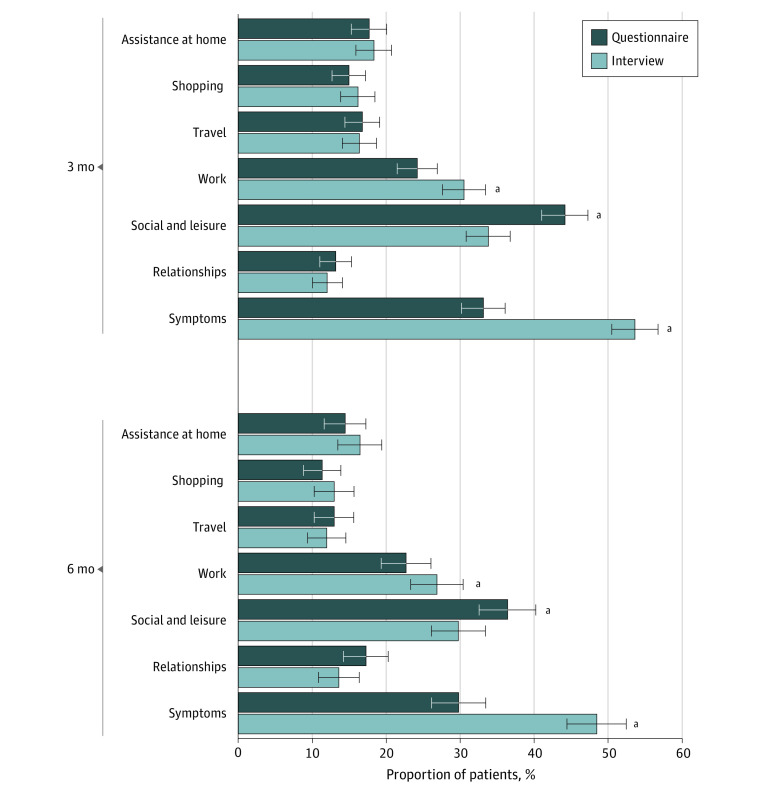
Problems and Limitations After Traumatic Brain Injury as Recorded on Subsections of the Glasgow Outcome Scale–Extended Questionnaire and Interview at 3-Month and 6-Month Follow-up Whiskers indicate 95% CIs. ^a^Difference significant at *P* < .01.

Because there were differences in recorded symptoms, we analyzed the data including all reported symptoms on the questionnaire (ie, not only the symptoms that were judged by the respondent to interfere with daily life). Agreement between the interview and questionnaire for symptoms increased to a κ of 0.56 (95% CI, 0.51-0.61) at 3 months and 0.50 (95% CI, 0.43-0.57) at 6 months. With use of this scoring, a similar proportion of patients had symptoms on the interview (524 [53.6%]) and on the questionnaire (501 [51.2%]) at 3 months (*P* = .13), whereas a smaller proportion of patients had symptoms on the interview (291 [48.4%]) than on the questionnaire (351 [58.4%]) at 6 months (*P* < .001).

### Comparative Validity

Correlations between variables are shown in Table 4. Correlations of the GOSE interview and questionnaire outcomes with baseline variables were strongest for the Glasgow Coma Scale score (interview: ρ, 0.42; questionnaire: ρ, 0.44) and the Injury Severity Score (interview: ρ, −0.43; questionnaire: ρ, −0.47) and weakest for pupil reactivity (interview: ρ, −0.18; questionnaire: ρ, −0.16) and age (interview: ρ, −0.12; questionnaire: ρ, −0.06). Correlations between the GOSE and other outcomes were generally stronger than those between the GOSE and baseline factors. The strongest correlations were between the GOSE and the SF-36v2 role-physical subscale (interview: ρ, 0.63; questionnaire: ρ, 0.65), the Physical Component Summary (interview: ρ, 0.56; questionnaire: ρ, 0.55), and the Quality of Life after Brain Injury Scale’s daily life and autonomy subscale (interview: ρ, 0.58; questionnaire: ρ, 0.59). Associations with baseline factors (ρ, –0.13 to 0.42) and patient-reported outcomes (ρ, 0.29 to 0.65) were similar between the interview and the questionnaire, and none of the differences reached a significance level of *P* < .01.

**Table 4.** Spearman Correlations Between GOSE Ratings, Baseline Factors, and 6-Month Outcomes and Differences Between Correlations^a^

Factor	Data points, No.	Spearman correlation, ρ^b^		Difference (95% CI)	*P* value
		GOSE interview	GOSE questionnaire		
Baseline factor					
Age	628	−0.12	−0.06	−0.06 (−0.13 to −0.01)	.03
GCS score	591	0.42	0.44	−0.02 (−0.07 to 0.03)	.47
Pupillary reactivity	579	−0.18	−0.16	−0.02 (−0.08 to 0.04)	.54
Total ISS	618	−0.43	−0.47	0.04 (−0.01 to 0.10)	.11
Head and neck AIS score	628	−0.47	−0.49	0.02 (−0.03 to 0.06)	.54
Major extracranial injury	628	−0.24	−0.30	0.06 (0.01 to 0.11)	.01
6-mo Outcome					
SF-36v2					
Physical functioning	497	0.53	0.50	0.03 (−0.03 to 0.10)	.28
Role-physical	495	0.63	0.65	−0.02 (−0.08 to 0.03)	.43
Pain	495	0.33	0.31	0.02 (−0.04 to 0.08)	.58
General health	497	0.41	0.36	0.05 (−0.01 to 0.10)	.14
Social functioning	495	0.49	0.52	−0.03 (−0.08 to 0.03)	.35
Role-emotional	495	0.44	0.44	<0.01 (−0.06 to 0.07)	.96
Energy and fatigue	492	0.43	0.42	0.01 (−0.04 to 0.07)	.58
Mental health	492	0.34	0.32	0.02 (−0.04 to 0.07)	.51
MCS score	491	0.36	0.36	<0.01 (−0.06 to 0.06)	.91
PCS score	491	0.56	0.55	0.01 (−0.05 to 0.07)	.71
QOLIBRI					
Cognition	483	0.44	0.40	0.04 (−0.02 to 0.10)	.17
Self	483	0.39	0.37	0.02 (−0.03 to 0.08)	.43
Daily life and autonomy	483	0.58	0.59	−0.01 (−0.06 to 0.05)	.91
Social relationships	483	0.30	0.26	0.04 (−0.03 to 0.09)	.28
Emotions	484	0.28	0.29	−0.01 (−0.07 to 0.05)	.77
Physical problems	483	0.51	0.53	−0.02 (−0.08 to 0.03)	.38
Total	483	0.53	0.52	0.01 (−0.04 to 0.07)	.62
PHQ-9	493	−0.48	−0.48	<0.01 (−0.05 to 0.06)	.96
GAD-7	493	−0.35	−0.35	<0.01 (–0.06 to 0.06)	.97
RPQ	375	−0.54	−0.53	−0.01 (−0.08 to 0.07)	.83

Abbreviations: AIS, Abbreviated Injury Scale; GAD-7, Generalized Anxiety Disorder–7; GCS, Glasgow Coma Scale; GOSE, Glasgow Outcome Scale–Extended; ISS, Injury Severity Score; MCS, Mental Component Summary; PCS, Physical Component Summary; PHQ-9, Patient Health Questionnaire–9 depression scale; QOLIBRI, Quality of Life After Brain Injury; RPQ, Rivermead Post-Concussion Symptoms Questionnaire; SF-36v2, 36-Item Short Form Health Survey, version 2.

^a^ The 95% CIs and probabilities were obtained from a percentile bootstrap method.

^b^ Correlations were significant at 2-tailed *P* < .01 except for the correlation of age with GOSE questionnaire at baseline.

## Discussion

### Overall Ratings

Overall, the GOSE scores from interviews and questionnaires were in good agreement. The GOSE consists of a hierarchy of broad categories, and thus, many individuals are unambiguously assessed as being in a particular category. Cases assessed near borderlines were associated with more uncertainty.^10^ Disagreement between the interview and questionnaire scores by 1 category was common and suggests that borderlines may be an important factor in differences between the 2 approaches. Some cases of TBI represent a challenge for global outcome assessment and may lead to large discrepancies. These challenging cases include those in individuals with preexisting limitations, which can mask any post-TBI changes.^10^ Consistent with this, more disagreement between the questionnaire and interview occurred in the context of preexisting systemic disease in the present study. However, large discrepancies between the 2 formats were found in only 5.8% of cases at 3 months and 4.0% at 6 months.

Guidelines for interviewing allow the assessor to use judgment to move the rating to a higher or lower category than indicated by the responses recorded.^9^ We found that interviewers were using such discretion, and this was particularly prominent for individuals who potentially had outcomes regarded as unfavorable. Consistent with this, when outcomes were dichotomized at the cut point between upper severe disability and lower moderate disability, the interviews at 3 months showed fewer ratings of an unfavorable outcome than did the questionnaires. A judgment to assign a higher category may have been made because later parts of the interview are inconsistent with dependency (eg, the person is back at work). However, it may also indicate bias on the part of interviewers toward assigning particular outcomes.

### Subsections of the GOSE

Levels of agreement on individual sections of the GOSE were highest for objective aspects of functioning such as independence in activities of daily living and lowest for subjective aspects such as TBI symptoms and personal relationships. Judgments about ability to return to participation in work and social and leisure activities can be hypothetical when the individual is still recovering, and the differences between interview and questionnaire outcomes seemed to decrease as the time since the TBI increased. These may be areas where the interview allowed finer judgment, particularly in the first few months after injury.

Of note, we found that symptoms that interfere with daily life were less likely to be recorded on the questionnaire than by interviewers. The findings suggest that respondents may find it difficult to judge the association between TBI-related symptoms and daily functioning and may even be unaware of symptoms that are relevant.^31^ These observations are consistent with literature on mild TBI,^32,33^ in which symptom reports can vary substantially depending on the method of data collection. Some researchers have found that self-report questionnaires yield more reports of symptoms than do interviews,^32^ whereas other researchers have found the opposite.^33^

Concerns have been raised about the use of patient reports in TBI studies, particularly that patient reports may suggest an overly optimistic perspective of recovery owing to lack of awareness.^34^ However, we did not find this to be true overall. To clarify issues of informant reliability, it would be useful to conduct further research in which self-awareness was examined directly.

### Comparative Validity

The associations found between the GOSE ratings and other factors were as expected from previous research.^35,36,37^ A key novel finding of this study was the similarity in the strength of the correlations between each of the 2 assessment methods and other variables. The expectation that stronger correlations would be found between interview ratings and baseline factors and between questionnaire scores and patient-reported outcomes was not shown. The concordance in the associations implies that the core information collected from interviews and questionnaires concerning global outcomes after TBI was similar. This conclusion is consistent with the good alignment that has been reported between prognostic models based on questionnaire or interview outcomes.^38^

This study found that there was not a definitive advantage of interviewing. In a single-center study with a limited number of data collectors, one may expect interviews to be superior to questionnaires for reasons already stated. However, in a large-scale study such as the CENTER-TBI project, the involvement of multiple interviewers may introduce additional variability in the assessment. Thus, the potential advantage given by interviews may be cancelled by interrater differences. To achieve benefit from interviewing, multicenter studies may need to address interrater differences systematically, for example, by regular and repeated training and by undertaking central monitoring of individual assessments.^39,40^

Investigators may wish to use the 2 formats (questionnaires and interviews) as alternative methods of data collection. The results of the current study support combining ratings from these separate sources with the caveat that some additional variation may be introduced. The study’s findings indicate that amendments to scoring may help to align the methods further. Investigators using questionnaires may also consider using interviews to obtain additional information concerning specific individuals. For example, individuals with a need for assistance in only 1 area (eg, home, shopping, or travel) would potentially be on the borderline for independence and might be followed up by interview.

The GOSE questionnaire was originally designed for postal administration and is readily adaptable for use as an online instrument or a smartphone app. The GOSE interview has been applied in conditions other than TBI, including stroke, cardiac arrest, and multiple trauma.^41,42,43^ The instrument could potentially be appropriately reworded to assess the long-term neurological consequences of other conditions that have an acute onset and before-and-after states, including sepsis and other illnesses that may manifest long-term neurological symptoms. Validation would be necessary and could open the way for large-scale data collection for a variety of conditions for which chronic neurological outcomes are underresearched.

### Limitations

This study has limitations. The comparison of interviews with questionnaires was a planned analysis of the CENTER-TBI project but was not an experimental design. Information about baseline factors or scores on other outcome assessments was not masked to investigators. Furthermore, systematic comparisons between different modes of data collection (ie, telephone vs in-person interviews or patients vs other informants) were not possible because of either limited numbers of cases or confounding with outcome distributions. Prospective studies are needed to compare modes of data collection. The study also excluded patients with severe preexisting neurological conditions, and this limits the generalizability of the findings to these patients; interviews may be more able to disentangle the association between complex preinjury conditions and TBI outcomes.

## Conclusions

In this cohort study, GOSE ratings of outcomes for TBI that were obtained from questionnaires and interviews had good overall agreement. We found some disagreement between GOSE categories as well as differences in the percentages of post-TBI problems recorded by each method. We also found that interviewers used judgment in their overall ratings. However, any differences in the ratings did not translate into differences in the validity of the assessments. In this large-scale, multicenter study, interviews did not seem to offer substantial advantages compared with central scoring of information collected directly from patients and caregivers using a questionnaire. These findings support the use of questionnaires in studies in which this form of contact may offer substantial practical advantages compared with interviews.

## Notes

Supplement 1. **eFigure.** Participant Selection Flowchart **eTable 1.** Comparison of Overall Ratings From the GOSE Structured Interview and Questionnaire at 3 and 6 Months After Injury **eTable 2.** Levels of Concordance Between Overall Scores for GOSE Structured Interview and GOSE Questionnaire for the Full Sample and Subgroups at 3 and 6 Months After Injury **eTable 3.** Dichotomization of Outcomes by Interviews and Questionnaires **eTable 4.** Comparison Between GOSE Ratings Assigned by Interviewers and Ratings From Central Scoring Based on Recorded Responses **eTable 5.** 2 × 2 Tables for Agreement Between Individual Sections of the GOSE Interview and Questionnaire **eAppendix.** Glasgow Outcome Scale–Extended Questionnaire and Scoring Key

Supplement 2.
Collaborative European NeuroTrauma Effectiveness Research in TBI (CENTER-TBI) Participants and Investigators
