# Supplementary material for: Questionnaires vs Interviews for the Assessment of Global Functional Outcomes After Traumatic Brain Injury
Source: JAMA Netw Open. 2021 Nov 11;4(11):e2134121. doi: 10.1001/jamanetworkopen.2021.34121 (PMC8586906; doi:10.1001/jamanetworkopen.2021.34121)
Supplement: Supplement 1. — eFigure. Participant Selection Flowchart eTable 1. Comparison of Overall Ratings From the GOSE Structured Interview and Questionnaire at 3 and 6 Months After Injury eTable 2. Levels of Concordance Between Overall Scores for GOSE Structured Interview and GOSE Questionnaire for the Full Sample and Subgroups at 3 and 6 Months After Injury eTable 3. Dichotomization of Outcomes by Interviews and Questionnaires eTable 4. Comparison Between GOSE Ratings Assigned by Interviewers and Ratings From Central Scoring Based on Recorded Responses eTable 5. 2 × 2 Tables for Agreement Between Individual Sections of the GOSE Interview and Questionnaire eAppendix. Glasgow Outcome Scale–Extended Questionnaire and Scoring Key [file jamanetwopen-e2134121-s001.pdf]

# Supplementary Online Content

Horton L, Rhodes J, Menon DK, Maas AIR, Wilson L; Collaborative European NeuroTrauma Effectiveness Research in TBI (CENTER-TBI) Participants and Investigators. Questionnaires vs interviews for the assessment of global functional outcomes after traumatic brain injury. *JAMA Netw Open*. 2021;4(11):e2134121. doi:10.1001/jamanetworkopen.2021.34121

**eFigure.** Participant Selection Flowchart

**eTable 1.** Comparison of Overall Ratings From the GOSE Structured Interview and Questionnaire at 3 and 6 Months After Injury

**eTable 2.** Levels of Concordance Between Overall Scores for GOSE Structured Interview and GOSE Questionnaire for the Full Sample and Subgroups at 3 and 6 Months After Injury

**eTable 3.** Dichotomization of Outcomes by Interviews and Questionnaires

**eTable 4.** Comparison Between GOSE Ratings Assigned by Interviewers and Ratings From Central Scoring Based on Recorded Responses

**eTable 5.** 2 × 2 Tables for Agreement Between Individual Sections of the GOSE Interview and Questionnaire

**eAppendix.** Glasgow Outcome Scale—Extended Questionnaire and Scoring Key

This supplementary material has been provided by the authors to give readers additional information about their work.

© 2021 Horton L et al. *JAMA Network Open*.

eFigure. Participant selection flowchart.

Total CENTER-TBI
core study sample (*n*=4509)

Aged
< 16 years (*n*=149)

Date of death within 6 months
(*n*=471)

Withdrawn from study
within 6 months *(n*=198)

**Eligible sample (*n*=3691)**
(surviving adult patients eligible
for follow-up at 3 months
and 6 months after injury)

Patients assessed on the
GOSE at 3 months (*n*=2660)

GOSE assessments at 3
months:
- Interview (n=1986)
- Questionnaire (n=1750)

Patients assessed using both
the GOSE interview and
questionnaire at 3 months:
1096

GOSEs
not completed
within 3-week
interval (n=112)

**3-month sample (*n*=994)**
(Patients assessed at 3 months using the
GOSE interview and questionnaire
within a 3-week interval)

Patients assessed on the
GOSE at 6 months *(n*=2604)

GOSE assessments at 6
months:
- Interview (n=2165)
- Questionnaire (n=977)

Total no. patients assessed
using the GOSE interview
and questionnaire at 6
months: 678

Total no. GOSE
not completed
within 3-week
interval (n=50)

**6-month sample (*n*=628)**
(Patients assessed at 6 months using the
GOSE interview and questionnaire within a 3-
week interval)

© 2021 Horton L et al. *JAMA Network Open*.

eTable 1. Comparison of overall ratings from the GOSE structured interview and questionnaire at 3 and 6 months after injury.

| 3-month structured interview | 3-month questionnaire | | | | | | Totals (%) |
|---|---|---|---|---|---|---|---|
| | Lower SD/VS | Upper SD | Lower MD | Upper MD | Lower GR | Upper GR | |
| **Lower SD/VS** | **73** | 11 | 2 | 0 | 2 | 2 | 90 (9%) |
| **Upper SD** | 16 | **36** | 8 | 2 | 6 | 8 | 76 (8%) |
| **Lower MD** | 9 | 22 | **44** | 8 | 11 | 10 | 104 (11%) |
| **Upper MD** | 2 | 21 | 21 | **45** | 30 | 27 | 146 (15%) |
| **Lower GR** | 3 | 17 | 14 | 21 | **67** | 137 | 259 (26%) |
| **Upper GR** | 3 | 2 | 3 | 9 | 32 | **270** | 319 (32%) |
| **Totals (%)** | 106 (11%) | 109 (11%) | 92 (9%) | 85 (9%) | 148 (15%) | 454 (46%) | 994 (100%) |

| 6-month structured interview | 6-month questionnaire | | | | | | Totals (%) |
|---|---|---|---|---|---|---|---|
| | Lower SD/VS | Upper SD | Lower MD | Upper MD | Lower GR | Upper GR | |
| **Lower SD/VS** | **54** | 3 | 0 | 0 | 1 | 1 | 59 (9%) |
| **Upper SD** | 3 | **15** | 1 | 3 | 3 | 5 | 30 (5%) |
| **Lower MD** | 0 | 11 | **51** | 4 | 4 | 6 | 76 (12%) |
| **Upper MD** | 1 | 7 | 16 | **34** | 17 | 15 | 90 (14%) |
| **Lower GR** | 1 | 5 | 4 | 13 | **51** | 92 | 166 (26) |
| **Upper GR** | 0 | 1 | 1 | 4 | 19 | **182** | 207 (33%) |
| **Totals (%)** | 59 (9%) | 42 (7%) | 73 (12%) | 58 (9%) | 95 (15%) | 301 (48%) | 628 (100%) |

Abbreviations: SD = severe disability, VS = vegetative state, MD = moderate disability, GR = good recovery

© 2021 Horton L et al. *JAMA Network Open*.

eTable 2. Levels of concordance between overall scores for GOSE structured interview and GOSE questionnaire for the full sample and subgroups at 3 and 6 months after injury.

| | **3-month GOSE assessments** $\kappa_w$ (95% CIs) | **6-month GOSE assessments** $\kappa_w$ (95% CIs) |
|---|---|---|
| **Full sample** | 0.76 (0.73-0.80) (n=994) | 0.82 (0.78-0.86) (n=628) |
| **Pre-injury health status** | | |
| Healthy (ASAP = 1) | 0.81 (0.77-0.85) (n=592) | 0.86 (0.83-0.90) (n=367) |
| Pre-existing systemic disease (ASAP 2 to 4) | 0.70 (0.63-0.76) (n=389) | 0.75 (0.67-0.83) (n=247) |
| **Major extra-cranial injury** | | |
| Absent | 0.73 (0.68-0.78) (n=643) | 0.75 (0.68-0.82) (n=424) |
| Present | 0.78 (0.73-0.83) (n=351) | 0.87 (0.83-0.91) (n=204) |
| **Highest level of education** | | |
| Primary | 0.79 (0.68-0.90) (n=90) | 0.78 (0.64-0.92) (n=65) |
| Secondary or above | 0.77 (0.73-0.81) (n=904) | 0.81 (0.77-0.86) (n=563) |

© 2021 Horton L et al. *JAMA Network Open*.

eTable 3. Dichotomization of outcomes by interviews and questionnaires.

**3-month**

| | | Questionnaire | | Total |
|---|---|---|---|---|
| | | Unfavourable | Favourable | |
| Interview | Unfavourable | 136 | 30 | 166 |
| | Favourable | 79 | 749 | 828 |
| | Total | 215 | 779 | 994 |

**6-month**

| | | Questionnaire | | Total |
|---|---|---|---|---|
| | | Unfavourable | Favourable | |
| Interview | Unfavourable | 75 | 14 | 89 |
| | Favourable | 26 | 513 | 539 |
| | Total | 101 | 527 | 628 |

'Unfavourable' = Upper Severe Disability or worse; 'favourable' = Lower Moderate Disability or better

© 2021 Horton L et al. *JAMA Network Open*.

eTable 4. Comparison between GOSE ratings assigned by interviewers and ratings from central scoring based on recorded responses. The table shows the number (percentage) of ratings by interviewers that were in a higher category (less disabled), in agreement, or in a lower category (more disabled) than assigned by the central scoring. Comparisons are for interviews conducted at 3 months (n=980) and 6 months (n=603). GOSE categories are from central scoring.

| Central score | Interviewer rating in comparison to central scoring | | |
|---|---|---|---|
| **3-month** | More disabled | In agreement | Less disabled |
| GOSE 3 | 0 (0%) | 54 (86%) | 9 (14%) |
| GOSE 4 | 1 (2%) | 22 (40%) | 32 (58%) |
| GOSE 5 | 0 (0%) | 53 (91%) | 5 (9%) |
| GOSE 6 | 3 (5%) | 54 (82%) | 9 (14%) |
| GOSE 7 | 18 (15%) | 98 (80%) | 7 (6%) |
| GOSE 8 | 59 (25%) | 179 (75%) | 0 (0%) |
| **6-month** | More disabled | In agreement | Less disabled |
| GOSE 3 | 0 (0%) | 84 (69%) | 38 (31%) |
| GOSE 4 | 2 (2%) | 51 (50%) | 50 (49%) |
| GOSE 5 | 1 (1%) | 65 (78%) | 17 (21%) |
| GOSE 6 | 9 (7%) | 91 (70%) | 30 (23%) |
| GOSE 7 | 20 (10%) | 156 (80%) | 19 (10%) |
| GOSE 8 | 71 (21%) | 276 (80%) | 0 (0%) |

© 2021 Horton L et al. *JAMA Network Open*.

eTable 5. 2 x 2 tables for agreement between individual sections of the GOSE interview and questionnaire.

| | n | I-/Q- | I-/Q+ | I+/Q- | I+/Q+ |
|---|---|---|---|---|---|
| **3-month** | | | | | |
| Assistance at home | 979 | 759 | 41 | 47 | 132 |
| Shopping | 979 | 801 | 20 | 32 | 126 |
| Travel | 979 | 789 | 30 | 26 | 134 |
| Work | 964 | 642 | 28 | 89 | 205 |
| Social & leisure | 977 | 502 | 145 | 44 | 286 |
| Relationships | 973 | 797 | 59 | 48 | 69 |
| Symptoms | 978 | 416 | 38 | 238 | 286 |
| **6-month** | | | | | |
| Assistance at home | 603 | 489 | 15 | 27 | 72 |
| Shopping | 602 | 512 | 12 | 22 | 56 |
| Travel | 603 | 512 | 19 | 13 | 59 |
| Work | 600 | 422 | 17 | 42 | 119 |
| Social & leisure | 602 | 351 | 72 | 32 | 147 |
| Relationships | 603 | 471 | 50 | 28 | 54 |
| Symptoms | 601 | 278 | 32 | 144 | 147 |

I- = no limitation recorded on interview; I+ = limitation recorded on interview
Q- = no limitation recorded on questionnaire; Q+ = limitation recorded on questionnaire

© 2021 Horton L et al. *JAMA Network Open*.

eAppendix. Glasgow Outcome Scale - Extended questionnaire and scoring key.

© 2021 Horton L et al. *JAMA Network Open*.

# Glasgow Outcome Scale - Extended Questionnaire

**These questions are to do with changes in your lifestyle since your injury. There are also some questions about how things were before the injury. The questions can be answered by you, or by a close relative or friend, or by you both together. We are interested in the recovery you have made up to now.**

Injured person's name: ___________________________________ Today's date: ___________

Person who filled out this form:
Patient alone☐ Relative or friend or carer alone☐ Patient and relative, friend or carer together ☐

The injured person is:
Out of hospital☐ In hospital or residential care☐

**Please answer each question by ticking one box ☑ which is true for you.**

---

1. Before the injury were you able to look after yourself at home? Yes ☐ No ☐

2. As a result of your injury do you now need someone to help look after you at home? **(please tick ☑ one box)**

I do not need help or supervision in the home ☐

I need some help in the home, but not every day ☐

I need help in the home every day, but I could look after myself for at least 8 hours if necessary ☐

I could not look after myself for 8 hours during the day ☐

I need help in the home, but not because of the injury ☐

---

3. Before the injury were you able to buy things at shops without help? Yes ☐ No ☐

4. As a result of your injury do you now need help to buy things at shops? **(please tick ☑ one box)**

I do not need help to shop ☐

I need some help, but I can go to local shops on my own ☐

I need help to shop even locally, or I cannot shop at all ☐

I need help to shop, but not because of the injury ☐

---

© 2021 Horton L et al. *JAMA Network Open*.

5. Before the injury were you able to travel without help? Yes ☐ No ☐

6. As a result of your injury do you now need help to travel? **(please tick ☑ one box)**

I do not need help to travel ☐

I need some help, but can travel locally on my own (e.g. by arranging a taxi) ☐

I need help to travel even locally, or I cannot travel at all ☐

I need help to travel but not because of the injury ☐

7. Employment before the injury: **(please tick ☑ one box)**

Working ☐ Looking for work ☐

Looking after family ☐ Studying as a student ☐

Retired ☐ None of these (e.g. unfit for work) ☐

8. As a result of your injury has there been a change in your ability to work? (or to study if you were a student; or to look after your family) **(please tick ☑ one box)**

I still do the same work ☐

I still do the same work, but have some problems (e.g. tiredness, lack of concentration). ☐

I still work, but at a reduced level (e.g. change from full-time to part-time, or change in level of responsibility) ☐

I am unable to work, or only able to work in sheltered workshop ☐

My ability to work has changed, but not because of the injury ☐

9. Before the injury did you take part in regular social and leisure activities outside home (at least once a week)? Yes ☐ No ☐

Social and leisure activities include: going out to a pub or club, visiting friends, going to the cinema or bingo, going out for a walk, attending a football match, taking part in sport.

10. As a result of your injury has there been a change in your ability to take part in social and leisure activities outside home? **(please tick ☑ one box)**

I take part about as often as before (the activities may be different from before) ☐

I take part a bit less, but at least half as often ☐

I take part much less, less than half as often ☐

I do not take part at all ☐

My ability to take part has changed for some other reason, not because of the injury ☐

© 2021 Horton L et al. *JAMA Network Open*.

11. Before the injury did you have any problems in getting on with friends or relatives?

Yes ☐ No ☐

12. As a result of your injury are there now problems in how you get on with friends or relatives? **(please tick ☑ one box)**

- Things are still much the same ☐
- There are occasional problems (less than once a week) ☐
- There are frequent problems (once a week or more) ☐
- There are constant problems (problems every day) ☐
- There are problems for some other reason, not because of the injury ☐

13. Are there any other problems resulting from your injury which have interfered with your daily life over the past week? (Problems sometimes reported after head injury: **headaches, dizziness, tiredness, sensitivity to noise or light, slowness, memory failures, and concentration problems**.) **(please tick ☑ one box)**

- I have no current problems ☐
- I have some problems, but these do not interfere with my daily life ☐
- I have some problems, and these have affected my daily life ☐
- I have some problems for other reasons, not because of the head injury ☐

14. Before the injury were similar problems present? **please tick ☑ one box)**

- I had no problems before, I had minor problems ☐
- I had similar problems before ☐

Are there any other comments that you would like to make?
(Please continue overleaf if you wish)

GOSE 8-point. Copyright © Lindsay Wilson and Graham Teasdale 2002. All rights reserved

© 2021 Horton L et al. *JAMA Network Open*.

Extended Glasgow Outcome Scale Questionnaire for completion by patients, relatives, carer

Key for scoring 8-point Extended Scale

| | |
|---|---|
| Upper GR | Upper Good Recovery |
| Lower GR | Lower Good Recovery |
| Upper MD | Upper Moderate Disability |
| Lower MD | Lower Moderate Disability |
| Upper SD | Upper Severe Disability |
| Lower SD | Lower Severe Disability |

SD* Severely Disabled pre-injury, or for some other reason not related to head injury

Instructions for rating outcome

The rating is the lowest outcome category indicated by the person's responses, but this may be modified in specific ways by responses to the pre-injury problems questions:
Questions 1 to 6 concern ability to live independently; people who were dependent pre-injury, or dependent for some reason other than head injury can be rated as SD* to distinguish them .
Questions 7- 14 concern ability to resume social roles in different areas of life; if the person had pre-injury limitations/problems in a particular area, or problems not related to head injury then the person is not rated on the question concerning this area (i.e. this area is ignored in the rating).

If the information is incomplete or inconsistent the rater may need to make a judgement concerning the overall rating (see Notes at end).

The following list shows the outcome category associated with each response, and the procedure for dealing with pre-injury problems or limitations. The responses for each of the main post-injury questions are given a letter here (a) – (e) in the order that they appear on the questionnaire.

Q1 Pre-injury independence in home
Yes
No = SD*
(If they tick (a) or (b) for Q2 below then do not rate as SD*)

Q2 Independence in home
(a) Fully independent
(b) Independent for more than 24 hrs (treated as independent)
(c) Dependent 8 - 24 hrs = Upper SD
(d) Dependent < 8 hrs = Lower SD
(e) Dependent for other reason = SD*

Q3 Pre-injury independence in shopping
Yes
No = SD*

© 2021 Horton L et al. *JAMA Network Open*.

(Note:  if they tick (a) or (b) for Q4 below then do not rate as SD*)

Q4 Independence in shopping
(a) Fully independent
(b) Independent for local shopping
(c) Dependent = Upper SD
(d) Dependent for other reason = SD*

Q5 Pre-injury independence in travel
Yes
No = SD*
(Note:  if they tick (a) or (b) for Q5 below then do not rate as SD*)

Q6 Independence in travel
(a) Fully independent
(b) Independent for local travel
(c) Dependent = Upper SD
(d) Dependent for other reason = SD*

Q7 Pre-injury work
Working, looking for work, looking after family, studying:   Can be rated on Q8.
Retired, none of these (eg unfit for work):   Do not rate on Q8

Q8 Work
(a) Same level of work
(b) Same level of work, some problems = Lower GR
(c) Lower work capacity = Upper MD
(d) Unable to work = Lower MD
(e) Work affected for other reason = Do not rate on Q8

Q9 Regular pre-injury regular social and leisure activities
Yes
No = Do not rate on Q10

Q10 Social and leisure activities outside home
(a) Participate as often as before
(b) Participate at least half as often as before = Lower GR
(c) Participate less than half as often = Upper MD
(d) Unable to participate = Lower MD
(e) Participate less for some other reason = Do not rate on Q10

Q11 Pre-injury problems with family and friendships
(a)Yes = Do not rate on Q11
(b) No

Q12 Family and friendships
(a) Relationships same as before

© 2021 Horton L et al. *JAMA Network Open*.

(b) Occasional problems - less than weekly = Lower GR
(c) Frequent problems - once a week or more = Upper MD
(d) Constant problems - daily = Lower MD
(e) Problems for some other reason = Do not rate on Q11

Q13 Any other problems in daily life
(a) No problems = Upper GR
(b) Some problems, not affecting daily life = Upper GR
(c) Problems affecting daily life = Lower GR
(d) Problems for other reason = Rate as Upper GR

Q14 Pre-injury problems
(a) No problems, minor problems
(b) Similar problems = Rate as Upper GR

NOTES

GOS Questionnaires: Inconsistent responses and responses for which further information is desirable

(1) Respondent ticks more than one box. In these circumstances it is usually fair to take the lowest category (greatest disability) indicated by the response. However, this pattern of responding suggests that the person does not fully understand the questionnaire.

(2) Respondent omits responses to some questions. Because of the nature of the scoring system it is sometimes possible for items to be omitted and still to obtain a rating. However, if more than a few items are incomplete then this will not be possible.
The work question may be omitted if the person was retired or not working before injury. The rating can be based on the answers to other questions.

(3) Responses are inconsistent. It is inconsistent if a respondent indicates that they are dependent and then responds that they are able to work (even at a reduced level). Ideally further information will be obtained to clarify the situation, otherwise a judgement needs to be made about the likely level of disability. For example, in such a case one might infer that the need for help indicated in activities was not with activities critical to independence, and that the person should not be rated as SD.

(4) Additional information qualifies responses. Information volunteered at the end of questionnaire or beside responses may alter the interpretation of some responses. For example, it may be clear that some disability stems from a cause other than injury. In this case this additional information should be used in arriving at an overall judgement of outcome category.

References
Jennett, B., & Bond, M. (1975). Assessment of outcome after severe brain damage. A practical scale. Lancet, i, 480-484.

© 2021 Horton L et al. *JAMA Network Open*.

Jennett, B., Snoek, J., Bond, M. R., & Brooks, N. (1981). Disability after severe head injury: Observations on the use of the Glasgow Outcome Scale. Journal of Neurology, Neurosurgery, & Psychiatry, 44, 285-293.
Wilson, J. T. L., Pettigrew, L. E. L., & Teasdale, G. M. (1998). Structured interviews for the Glasgow Outcome Scale and Extended Glasgow Outcome Scale: Guidelines for their use. Journal of Neurotrauma, 15, 573-585.
Wilson J.T.L., Edwards P., Fiddes H. Stewart E., Teasdale G.M. (2002) Reliability of postal questionnaires for the Glasgow Outcome Scale. Journal of Neurotrauma, 19, 999-1006.

© 2021 Horton L et al. *JAMA Network Open*.
