# Supplementary material for: Questionnaires vs Interviews for the Assessment of Global Functional Outcomes After Traumatic Brain Injury
Source: JAMA Netw Open. 2021 Nov 11;4(11):e2134121. doi: 10.1001/jamanetworkopen.2021.34121 (PMC8586906; doi:10.1001/jamanetworkopen.2021.34121)
Supplement: Supplement 2. — Collaborative European NeuroTrauma Effectiveness Research in TBI (CENTER-TBI) Participants and Investigators [file jamanetwopen-e2134121-s002.pdf]

Supplemental Online Content: Nonauthor Collaborators

*Indicates required information. Only first name, last name, and suffix will appear in PubMed.

*Group Name(s): Collaborative European NeuroTrauma Effectiveness Research in TBI (CENTER-TBI) Participants and Investigators							
***First Name and Middle Initial(s)**	***Last Name**	***Suffix (eg, Jr, III)**	Academic Degrees	Institution	Location (city, state/province, country)	Role or Contribution, eg, chair, principal investigator	Group (if more than 1 Group listed in the byline) and/or Subgroup (eg, Steering Committee)
Russell L.	Gruen			College of Health and Medicine, Australian National University, Canberra, Australia	Canberra, Australia	CENTER-TBI Associated Participant	
Peter	Cameron			ANZIC Research Centre, Monash University, Department of Epidemiology and Preventive Medicine, Melbourne, Vitoria, Australia	Melbourne, Australia	CENTER-TBI Participant	
Emma	Donoghue			ANZIC Research Centre, Monash University, Department of Epidemiology and Preventive Medicine, Melbourne, Vitoria, Australia	Melbourne, Australia	CENTER-TBI Participant	
Dashiell	Gantner			ANZIC Research Centre, Monash University, Department of Epidemiology and Preventive Medicine, Melbourne, Vitoria, Australia	Melbourne, Australia	Oz ENTER	
Tony	Trapani			ANZIC Research Centre, Monash University, Department of Epidemiology and Preventive Medicine, Melbourne, Vitoria, Australia	Melbourne, Australia	Oz ENTER	
Shirley	Vallance			ANZIC Research Centre, Monash University, Department of Epidemiology and Preventive Medicine, Melbourne, Vitoria, Australia	Melbourne, Australia	Oz ENTER	

Supplemental Online Content: Nonauthor Collaborators

*Indicates required information. Only first name, last name, and suffix will appear in PubMed.

*First Name and Middle Initial(s)	*Last Name	*Suffix (eg, Jr, III)	Academic Degrees	Institution	Location (city, state/province, country)	Role or Contribution, eg, chair, principal investigator	Group (if more than 1 Group listed in the byline) and/or Subgroup (eg, Steering Committee)
Lynnette	Murray			ANZIC Research Centre, Monash University, Department of Epidemiology and Preventive Medicine, Melbourne, Vitoria, Australia	Melbourne, Australia	Oz ENTER	
Joanne	Brooker			Australian & New Zealand Intensive Care Research Centre, Department of Epidemiology and Preventive Medicine, School of Public Health and Preventive Medicine, Monash University, Melbourne, Australia	Melbourne, Australia	CENTER-TBI Participant	
Peter	Bragge			BehaviourWorks Australia, Monash Sustainability Institute, Monash University, Victoria, Australia	Melbourne, Australia	CENTER-TBI Participant	
Jeffrey	Rosenfeld			National Trauma Research Institute, The Alfred Hospital, Monash University, Melbourne, Victoria, Australia	Melbourne, Australia	CENTER-TBI Participant	
Jamie D.	Cooper			School of Public Health & PM, Monash University and The Alfred Hospital, Melbourne, Victoria, Australia	Melbourne, Australia	CENTER-TBI Participant + Investigator	
Raimund	Helbok			Department of Neurology, Neurological Intensive Care Unit, Medical University of Innsbruck, Innsbruck, Austria	Innsbruck, Austria	CENTER-TBI Investigator	

*Indicates required information. Only first name, last name, and suffix will appear in PubMed.

*First Name and Middle Initial(s)	*Last Name	*Suffix (eg, Jr, III)	Academic Degrees	Institution	Location (city, state/province, country)	Role or Contribution, eg, chair, principal investigator	Group (if more than 1 Group listed in the byline) and/or Subgroup (eg, Steering Committee)
Ronny	Beer			Department of Neurology, Neurological Intensive Care Unit, Medical University of Innsbruck, Innsbruck, Austria	Innsbruck, Austria	CENTER-TBI Investigator	
Herbert	Schoechl			Department of Anaesthesiology and Intensive Care, AUVA Trauma Hospital, Salzburg, Austria	Salzburg, Austria	CENTER-TBI Associated Participant	
Martin	Rusnák			International Neurotrauma Research Organisation, Vienna, Austria	Vienna, Austria	CENTER-TBI Participant	
Elisabeth	Schwendenwein			Trauma Surgery, Medical University Vienna, Vienna, Austria	Vienna, Austria	CENTER-TBI Investigator	
Anna	Antoni			Trauma Surgery, Medical University Vienna, Vienna, Austria	Vienna, Austria	CENTER-TBI Investigator	
Véronique	De Keyser			Department of Neurosurgery, Antwerp University Hospital and University of Antwerp, Edegem, Belgium	Edegem, Belgium	CENTER-TBI Participant + Investigator	
Tomas	Menovsky			Department of Neurosurgery, Antwerp University Hospital and University of Antwerp, Edegem, Belgium	Edegem, Belgium	CENTER-TBI Participant + Investigator	
Dominique	Van Praag			Psychology Department, Antwerp University Hospital, Edegem, Belgium	Edegem, Belgium	CENTER-TBI Participant + Investigator	

*Indicates required information. Only first name, last name, and suffix will appear in PubMed.

***First Name and Middle Initial(s)**	***Last Name**	***Suffix (eg, Jr, III)**	Academic Degrees	Institution	Location (city, state/province, country)	Role or Contribution, eg, chair, principal investigator	Group (if more than 1 Group listed in the byline) and/or Subgroup (eg, Steering Committee)
Andrew I.R.	Maas			Department of Neurosurgery, Antwerp University Hospital and University of Antwerp, Edegem, Belgium	Edegem, Belgium	CENTER-TBI Participant + Investigator	
Gregory	Van der Steen			Department of Neurosurgery, Antwerp University Hospital and University of Antwerp, Edegem, Belgium	Edegem, Belgium	CENTER-TBI Participant	
Paul M.	Parizel			Department of Radiology, University of Antwerp, Edegem, Belgium	Edegem, Belgium	CENTER-TBI Participant + Investigator	
Thijs	Vande Vyvere			icoMetrix NV, Leuven, Belgium	Edegem, Belgium	CENTER-TBI Participant + Investigator	
Bart	Depreitere			Department of Neurosurgery, University Hospitals Leuven, Leuven, Belgium	Leuven, Belgium	CENTER-TBI Investigator	
Wim	Van Hecke			icoMetrix NV, Leuven, Belgium	Leuven, Belgium	CENTER-TBI Participant	
Jan	Verheyden			icoMetrix NV, Leuven, Belgium	Leuven, Belgium	CENTER-TBI Participant	
Benoit	Misset			Cyclotron Research Center , University of Liège, Liège, Belgium	Liège, Belgium	CENTER-TBI Participant + Investigator	
Didier	Ledoux			Cyclotron Research Center , University of Liège, Liège, Belgium	Liège, Belgium	CENTER-TBI Participant + Investigator	
Steven	Laureys			Cyclotron Research Center , University of Liège, Liège, Belgium	Liège, Belgium	CENTER-TBI Participant + Investigator	
Alexandre	Ghuysen			Emergency Department, CHU , Liège, Belgium	Liège, Belgium	CENTER-TBI Investigator	
Hugues	Maréchal			Intensive Care Unit, CHR Citadelle , Liège, Belgium	Liège, Belgium	CENTER-TBI Investigator	

*Indicates required information. Only first name, last name, and suffix will appear in PubMed.

***First Name and Middle Initial(s)**	***Last Name**	***Suffix (eg, Jr, III)**	Academic Degrees	Institution	Location (city, state/province, country)	Role or Contribution, eg, chair, principal investigator	Group (if more than 1 Group listed in the byline) and/or Subgroup (eg, Steering Committee)
Guy-Loup	Dulière			Intensive Care Unit, CHR Citadelle , Liège, Belgium	Liège, Belgium	CENTER-TBI Investigator	
Guoyi	Gao			Department of Neurosurgery, Shanghai Renji hospital, Shanghai Jiaotong University/school of medicine, Shanghai, China	Shanghai, China	CENTER-TBI Participant	
Ji-yao	Jiang			Department of Neurosurgery, Shanghai Renji hospital, Shanghai Jiaotong University/school of medicine, Shanghai, China	Shanghai, China	CENTER-TBI Participant	
Daniel	Kondziella			Departments of Neurology, Clinical Neurophysiology and Neuroanesthesiology, Region Hovedstaden Rigshospitalet, Copenhagen, Denmark	Copenhagen, Denmark	CENTER-TBI Investigator	
Martin	Fabricius			Departments of Neurology, Clinical Neurophysiology and Neuroanesthesiology, Region Hovedstaden Rigshospitalet, Copenhagen, Denmark	Copenhagen, Denmark	CENTER-TBI Participant + Investigator	
Rico Frederik	Schou			Department of Neuroanesthesia and Neurointensive Care, Odense University Hospital, Odense, Denmark	Odense, Denmark	CENTER-TBI Participant + Investigator	
Morten	Blaabjerg			Department of Neurology, Odense University Hospital, Odense, denmark	Odense, Denmark	CENTER-TBI Participant + Investigator	

*Indicates required information. Only first name, last name, and suffix will appear in PubMed.

*First Name and Middle Initial(s)	*Last Name	*Suffix (eg, Jr, III)	Academic Degrees	Institution	Location (city, state/province, country)	Role or Contribution, eg, chair, principal investigator	Group (if more than 1 Group listed in the byline) and/or Subgroup (eg, Steering Committee)
Christina	Rosenlund			Department of Neurosurgery, Odense University Hospital, Odense, Denmark	Odense, Denmark	CENTER-TBI Investigator	
Anna	Piippo-Karjalainen			Department of Neurosurgery, Helsinki University Central Hospital	Helsinki, Finland	CENTER-TBI Investigator	
Rahul	Raj			Department of Neurosurgery, Helsinki University Central Hospital	Helsinki, Finland	CENTER-TBI Investigator	
Matti	Pirinen			Institute for Molecular Medicine Finland, University of Helsinki, Helsinki, Finland	Helsinki, Finland	CENTER-TBI Participant	
Samuli	Ripatti			Institute for Molecular Medicine Finland, University of Helsinki, Helsinki, Finland	Helsinki, Finland	CENTER-TBI Participant	
Aarno	Palotie			Institute for Molecular Medicine Finland, University of Helsinki, Helsinki, Finland	Helsinki, Finland	CENTER-TBI Participant	
Peter	Ylén			VTT Technical Research Centre, Tampere, Finland	Tampere, Finland	CENTER-TBI Participant	
Jussi P.	Posti			Division of Clinical Neurosciences, Department of Neurosurgery and Turku Brain Injury Centre, Turku University Hospital and University of Turku, Turku, Finland	Turku, Finland	CENTER-TBI Participant + Investigator	

*Indicates required information. Only first name, last name, and suffix will appear in PubMed.

*First Name and Middle Initial(s)	*Last Name	*Suffix (eg, Jr, III)	Academic Degrees	Institution	Location (city, state/province, country)	Role or Contribution, eg, chair, principal investigator	Group (if more than 1 Group listed in the byline) and/or Subgroup (eg, Steering Committee)
Olli	Tenovuo			Division of Clinical Neurosciences, Department of Neurosurgery and Turku Brain Injury Centre, Turku University Hospital and University of Turku, Turku, Finland	Turku, Finland	CENTER-TBI Participant + Investigator	
Riikka	Takala			Perioperative Services, Intensive Care Medicine and Pain Management, Turku University Hospital and University of Turku, Turku, Finland.	Turku, Finland	CENTER-TBI Participant + Investigator	
Jean-François	Payen			Department of Anesthesiology & Intensive Care, University Hospital of Grenoble, Grenoble, France	Grenoble, France	CENTER-TBI Investigator	
Emmanuel	Vega			Department of Anesthesiology-Intensive Care, Lille University Hospital, Lille, France	Lille, France	CENTER-TBI Investigator	
Aurelie	Lejeune			Department of Anesthesiology-Intensive Care, Lille University Hospital, Lille, France	Lille, France	CENTER-TBI Investigator	
Gérard	Audibert			Department of Anesthesiology & Intensive Care, University Hospital Nancy, Nancy, France	Nancy, France	CENTER-TBI Investigator	
Vincent	Degos			Anesthesie-Réanimation, Assistance Publique – Hopitaux de Paris, Paris, France	Paris, France	CENTER-TBI Participant + Investigator	
Habib	Benali			Anesthesie-Réanimation, Assistance Publique – Hopitaux de Paris, Paris, France	Paris, France	CENTER-TBI Participant + Investigator	

*Indicates required information. Only first name, last name, and suffix will appear in PubMed.

***First Name and Middle Initial(s)**	***Last Name**	***Suffix (eg, Jr, III)**	Academic Degrees	Institution	Location (city, state/province, country)	Role or Contribution, eg, chair, principal investigator	Group (if more than 1 Group listed in the byline) and/or Subgroup (eg, Steering Committee)
Damien	Galanaud			Anesthesie-Réanimation, Assistance Publique – Hopitaux de Paris, Paris, France	Paris, France	CENTER-TBI Participant + Investigator	
Vincent	Perlbarg			Anesthesie-Réanimation, Assistance Publique – Hopitaux de Paris, Paris, France	Paris, France	CENTER-TBI Participant + Investigator	
Louis	Puybasset			Department of Anesthesiology and Critical Care, Pitié -Salpêtrière Teaching Hospital, Assistance Publique, Hôpitaux de Paris and University Pierre et Marie Curie, Paris, France	Paris, France	CENTER-TBI Participant + Investigator	
Philippe	Azouvi			Raymond Poincare hospital, Assistance Publique – Hopitaux de Paris, Paris, France	Paris, France	CENTER-TBI Participant	
Valerie	Legrand			VP Global Project Management CNS, ICON, Paris, France	Paris, France	CENTER-TBI Participant	
Claire	Dahyot-Fizelier			Intensive care Unit, CHU Poitiers, Poitiers, France	Poitiers, France	CENTER-TBI Investigator	
Rolf	Rossaint			Department of Anaesthesiology, University Hospital of Aachen, Aachen, Germany	Aachen, Germany	CENTER-TBI Associated participant + Investigator	
Mark Steven	Coburn			Department of Anesthesiology and Intensive Care Medicine, University Hospital Bonn, Bonn, Germany	Bonn, Germany	CENTER-TBI Investigator	

*Indicates required information. Only first name, last name, and suffix will appear in PubMed.

***First Name and Middle Initial(s)**	***Last Name**	***Suffix (eg, Jr, III)**	Academic Degrees	Institution	Location (city, state/province, country)	Role or Contribution, eg, chair, principal investigator	Group (if more than 1 Group listed in the byline) and/or Subgroup (eg, Steering Committee)
Ana	Kowark			Department of Anesthesiology, University Hospital RWTH Aachen, Germany	Aachen, Germany	CENTER-TBI Investigator	
Hans	Clusmann			Department of Neurosurgery, Medical Faculty RWTH Aachen University, Aachen, Germany	Aachen, Germany	CENTER-TBI Investigator	
Jens	Dreier			Interdisciplinary Neuro Intensive Care Unit , Charité – Universitätsmedizin Berlin, Berlin, Germany	Berlin, Germany	CENTER-TBI Participant + Investigator	
Stefan	Wolf			Interdisciplinary Neuro Intensive Care Unit , Charité – Universitätsmedizin Berlin, Berlin, Germany	Berlin, Germany	CENTER-TBI Participant + Investigator	
Peter	Vajkoczy			Neurologie, Neurochirurgie und Psychiatrie, Charité – Universitätsmedizin Berlin, Berlin, Germany	Berlin, Germany	CENTER-TBI Investigator	
Marc	Maegele			Cologne-Merheim Medical Center (CMMC), Department of Traumatology, Orthopedic Surgery and Sportmedicine, Witten/Herdecke University, Cologne, Germany	Cologne, Germany	CENTER-TBI Participant	
Johannes	Gratz			Department of Anesthesia, Critical Care and Pain Medicine, Medical University of Vienna	Vienna, Austria	CENTER-TBI Participant	

Supplemental Online Content: Nonauthor Collaborators

*Indicates required information. Only first name, last name, and suffix will appear in PubMed.

***First Name and Middle Initial(s)**	***Last Name**	***Suffix (eg, Jr, III)**	Academic Degrees	Institution	Location (city, state/province, country)	Role or Contribution, eg, chair, principal investigator	Group (if more than 1 Group listed in the byline) and/or Subgroup (eg, Steering Committee)
Nadine	Schäfer			Cologne-Merheim Medical Center (CMMC), Department of Traumatology, Orthopedic Surgery and Sportmedicine, Witten/Herdecke University, Cologne, Germany	Cologne, Germany	CENTER-TBI Participant	
Rolf	Lefering			Institute of Research in Operative Medicine (IFOM) , Witten/Herdecke University, Cologne, Germany	Cologne, Germany	CENTER-TBI Participant	
Amra	Čović			Institute of Medical Psychology and Medical Sociology, Universitätsmedizin Göttingen, Göttingen, Germany	Göttingen, Germany	CENTER-TBI Participant	
Nicole	von Steinbüchel			Institute of Medical Psychology and Medical Sociology, Universitätsmedizin Göttingen, Göttingen, Germany	Göttingen, Germany	CENTER-TBI Participant	
Silke	Schmidt			Department Health and Prevention, University Greifswald, Greifswald, Germany	Greifswald, Germany	CENTER-TBI Participant	
Monika	Bullinger			Department of Medical Psychology, Universitätsklinikum Hamburg-Eppendorf, Hamburg, Germany	Hamburg, Germany	CENTER-TBI Associated Participant	
Alexander	Younsi			Department of Neurosurgery, University Hospital Heidelberg, Heidelberg, Germany	Heidelberg, Germany	CENTER-TBI Investigator	

*Indicates required information. Only first name, last name, and suffix will appear in PubMed.

***First Name and Middle Initial(s)**	***Last Name**	***Suffix (eg, Jr, III)**	Academic Degrees	Institution	Location (city, state/province, country)	Role or Contribution, eg, chair, principal investigator	Group (if more than 1 Group listed in the byline) and/or Subgroup (eg, Steering Committee)
Andreas	Unterberg			Department of Neurosurgery, University Hospital Heidelberg, Heidelberg, Germany	Heidelberg, Germany	CENTER-TBI Participant + Investigator	
Julia	Mattern			Department of Neurosurgery, University Hospital Heidelberg, Heidelberg, Germany	Heidelberg, Germany	CENTER-TBI Participant	
Oliver	Sakowitz			Department of Neurosurgery, University Hospital Heidelberg, Heidelberg, Germany	Heidelberg, Germany	CENTER-TBI Participant	
Renan	Sanchez-Porras			Klinik für Neurochirurgie, Klinikum Ludwigsburg, Ludwigsburg, Germany	Ludwigsburg, Germany	CENTER-TBI Participant + Investigator	
Oliver	Sakowitz			Klinik für Neurochirurgie, Klinikum Ludwigsburg, Ludwigsburg, Germany	Ludwigsburg, Germany	CENTER-TBI Participant + Investigator	
Natascha	Perera			International Projects Management, ARTTIC, Munchen, Germany	Munchen, Germany	CENTER-TBI Participant	
Romuald	Beauvais			International Projects Management, ARTTIC, Munchen, Germany	Munchen, Germany	CENTER-TBI Participant	
Janos	Sandor			Division of Biostatistics and Epidemiology, Department of Preventive Medicine, University of Debrecen, Debrecen, Hungary	Debrecen, Hungary	CENTER-TBI Participant	

*Indicates required information. Only first name, last name, and suffix will appear in PubMed.

*First Name and Middle Initial(s)	*Last Name	*Suffix (eg, Jr, III)	Academic Degrees	Institution	Location (city, state/province, country)	Role or Contribution, eg, chair, principal investigator	Group (if more than 1 Group listed in the byline) and/or Subgroup (eg, Steering Committee)
Endre	Czeiter			Department of Neurosurgery, Medical School, University of Pécs, Hungary and Neurotrauma Research Group, János Szentágothai Research Centre, University of Pécs, Hungary	Pecs, Hungary	CENTER-TBI Participant + Investigator	
Andras	Buki			Department of Neurosurgery, Medical School, University of Pécs, Hungary and Neurotrauma Research Group, János Szentágothai Research Centre, University of Pécs, Hungary	Pecs, Hungary	CENTER-TBI Participant + Investigator	
Erzsébet	Ezer			Department of Anaesthesiology and Intensive Therapy, University of Pécs, Pécs, Hungary	Pécs, Hungary	CENTER-TBI Participant + Investigator	
Zoltán	Vámos			Department of Anaesthesiology and Intensive Therapy, University of Pécs, Pécs, Hungary	Pécs, Hungary	CENTER-TBI Participant + Investigator	
Béla	Melegh			Department of Medical Genetics, University of Pécs, Pécs, Hungary	Pécs, Hungary	CENTER-TBI Participant	
Viktória	Tamás			Department of Neurosurgery, University of Pécs, Pécs, Hungary	Pécs, Hungary	CENTER-TBI Participant + Investigator	
Abayomi	Sorinola			Department of Neurosurgery, University of Pécs, Pécs, Hungary	Pécs, Hungary	CENTER-TBI Participant + Investigator	

*Indicates required information. Only first name, last name, and suffix will appear in PubMed.

***First Name and Middle Initial(s)**	***Last Name**	***Suffix (eg, Jr, III)**	Academic Degrees	Institution	Location (city, state/province, country)	Role or Contribution, eg, chair, principal investigator	Group (if more than 1 Group listed in the byline) and/or Subgroup (eg, Steering Committee)
Noémi	Kovács			Hungarian Brain Research Program - Grant No. KTIA_13_NAP-A-II/8, University of Pécs, Pécs, Hungary	Pécs, Hungary	CENTER-TBI Participant + Investigator	
József	Nyirádi			János Szentágothai Research Centre, University of Pécs, Pécs, Hungary	Pécs, Hungary	CENTER-TBI Participant + Investigator	
Krisztina	Amrein			János Szentágothai Research Centre, University of Pécs, Pécs, Hungary	Pécs, Hungary	CENTER-TBI Participant + Investigator	
Pál	Barzó			Department of Neurosurgery, University of Szeged, Szeged, Hungary	Szeged, Hungary	CENTER-TBI Investigator	
Deepak	Gupta			Department of Neurosurgery, Neurosciences Centre & JPN Apex trauma centre, All India Institute of Medical Sciences, New Delhi-110029, India	Delhi, India	CENTER-TBI Associated participant + Investigator	
Leon	Levi			Department of Neurosurgery, Rambam Medical Center, Haifa, Israel	Haifa, Israel	CENTER-TBI Investigator	
Guy	Rosenthal			Department of Neurosurgery, Hadassah-hebrew University Medical center, Jerusalem, Israel	Jerusalem, Israel	CENTER-TBI Investigator	
Alex	Furmanov			Department of Neurosurgery, Hadassah-hebrew University Medical center, Jerusalem, Israel	Jerusalem, Israel	CENTER-TBI Investigator	

*Indicates required information. Only first name, last name, and suffix will appear in PubMed.

*First Name and Middle Initial(s)	*Last Name	*Suffix (eg, Jr, III)	Academic Degrees	Institution	Location (city, state/province, country)	Role or Contribution, eg, chair, principal investigator	Group (if more than 1 Group listed in the byline) and/or Subgroup (eg, Steering Committee)
Costanza	Martino			Department of Anesthesia & Intensive Care,M. Bufalini Hospital, Cesena, Italy	Cesena, Italy	CENTER-TBI Investigator	
Luigi	Beretta			Department of Anesthesiology & Intensive Care, S Raffaele University Hospital, Milan, Italy	Milan, Italy	CENTER-TBI Investigator	
Maria Rosa	Calvi			Department of Anesthesiology & Intensive Care, S Raffaele University Hospital, Milan, Italy	Milan, Italy	CENTER-TBI Investigator	
Maria Luisa	Azzolini			Department of Anesthesiology & Intensive Care, S Raffaele University Hospital, Milan, Italy	Milan, Italy	CENTER-TBI Investigator	
Nino	Stocchetti			Department of Pathophysiology and Transplantation, Milan University, and Neuroscience ICU, Fondazione IRCCS Cà Granda Ospedale Maggiore Policlinico, Milano, Italy	Milan, Italy	CENTER-TBI Participant + Investigator + MC	
Emiliana	Calappi			Neuro ICU, Fondazione IRCCS Cà Granda Ospedale Maggiore Policlinico, Milan, Italy	Milan, Italy	CENTER-TBI Participant + Investigator	
Tommaso	Zoerle			Neuro ICU, Fondazione IRCCS Cà Granda Ospedale Maggiore Policlinico, Milan, Italy	Milan, Italy	CENTER-TBI Participant + Investigator	

*Indicates required information. Only first name, last name, and suffix will appear in PubMed.

*First Name and Middle Initial(s)	*Last Name	*Suffix (eg, Jr, III)	Academic Degrees	Institution	Location (city, state/province, country)	Role or Contribution, eg, chair, principal investigator	Group (if more than 1 Group listed in the byline) and/or Subgroup (eg, Steering Committee)
Fabrizio	Ortolano			Neuro ICU, Fondazione IRCCS Cà Granda Ospedale Maggiore Policlinico, Milan, Italy	Milan, Italy	CENTER-TBI Participant + Investigator	
Marco	Carbonara			Neuro ICU, Fondazione IRCCS Cà Granda Ospedale Maggiore Policlinico, Milan, Italy	Milan, Italy	CENTER-TBI Participant + Investigator	
Alessio	Caccioppola			Neuro ICU, Fondazione IRCCS Cà Granda Ospedale Maggiore Policlinico, Milan, Italy	Milan, Italy	CENTER-TBI Participant + Investigator	
Giuseppe	Citerio			School of Medicine and Surgery, Università Milano Bicocca, Milano, Italy	Milano, Italy	CENTER-TBI Participant + Investigator + MC	
Alessia	Vargiolu			NeuroIntenisve Care Unit, Department of Anesthesia & Intensive Care Azienda Ospedaliera San Gerardo di Monza, Monza, Italy	Monza, Italy	CENTER-TBI Participant + Investigator	
Giuseppe	Citerio			NeuroIntenisve Care Unit, Department of Anesthesia & Intensive Care Azienda Ospedaliera San Gerardo di Monza, Monza, Italy	Monza, Italy	CENTER-TBI Participant + Investigator + MC	
Arturo	Chieregato			NeuroIntensive Care, Niguarda Hospital	Niguarda, Italy	CENTER-TBI Investigator	
Giorgio	Chevallard			NeuroIntensive Care, Niguarda Hospital	Niguarda, Italy	CENTER-TBI Investigator	

*Indicates required information. Only first name, last name, and suffix will appear in PubMed.

*First Name and Middle Initial(s)	*Last Name	*Suffix (eg, Jr, III)	Academic Degrees	Institution	Location (city, state/province, country)	Role or Contribution, eg, chair, principal investigator	Group (if more than 1 Group listed in the byline) and/or Subgroup (eg, Steering Committee)
Francesco	Della Corte			Department of Anesthesia & Intensive Care, Maggiore Della Carità Hospital, Novara, Italy	Novara, Italy	CENTER-TBI Investigator	
Francesca	Grossi			Department of Anesthesia & Intensive Care, Maggiore Della Carità Hospital, Novara, Italy	Novara, Italy	CENTER-TBI Investigator	
Sandra	Rossi			Department of Anesthesia & Intensive Care, Azienda Ospedaliera Università di Padova, Padova, Italy	Padova, Italy	CENTER-TBI Investigator	
Paolo	Persona			Department of Anesthesia & Intensive Care, Azienda Ospedaliera Università di Padova, Padova, Italy	Padova, Italy	CENTER-TBI Investigator	
Maurizio	Berardino			Department of Anesthesia & ICU, AOU Città della Salute e della Scienza di Torino - Orthopedic and Trauma Center, Torino, Italy	Torino, Italy	CENTER-TBI Investigator	
Simona	Cavallo			Department of Anesthesia & ICU, AOU Città della Salute e della Scienza di Torino - Orthopedic and Trauma Center, Torino, Italy	Torino, Italy	CENTER-TBI Investigator	
Malinka	Rambadagalla			Rezekne Hospital, Latvia	Rezekne, Latvia	CENTER-TBI Investigator	
Agate	Ziverte			Neurosurgery clinic, Pauls Stradins Clinical University Hospital, Riga, Latvia	Riga, Latvia	CENTER-TBI Investigator	
Lelde	Giga			Neurosurgery clinic, Pauls Stradins Clinical University Hospital, Riga, Latvia	Riga, Latvia	CENTER-TBI Investigator	

*Indicates required information. Only first name, last name, and suffix will appear in PubMed.

*First Name and Middle Initial(s)	*Last Name	*Suffix (eg, Jr, III)	Academic Degrees	Institution	Location (city, state/province, country)	Role or Contribution, eg, chair, principal investigator	Group (if more than 1 Group listed in the byline) and/or Subgroup (eg, Steering Committee)
Egils	Valeinis			Neurosurgery clinic, Pauls Stradins Clinical University Hospital, Riga, Latvia	Riga, Latvia	CENTER-TBI Investigator	
Rimantas	Vilcinis			Department of Neurosurgery, Kaunas University of Health Sciences, Kaunas, Lithuania	Kaunas, Lithuania	CENTER-TBI Investigator	
Tomas	Tamosuitis			Department of Neurosurgery, Kaunas University of technology and Vilnius University, Vilnius, Lithuania	Vilnius, Lithuania	CENTER-TBI Investigator	
Saulius	Rocka			Department of Neurosurgery, Kaunas University of technology and Vilnius University, Vilnius, Lithuania	Vilnius, Lithuania	CENTER-TBI Investigator	
Arminas	Ragauskas			Department of Neurosurgery, Kaunas University of technology and Vilnius University, Vilnius, Lithuania	Vilnius, Lithuania	CENTER-TBI Investigator	
Joukje	van der Naalt			Department of Neurology, University Medical Center Groningen, Groningen, Netherlands	Groningen, Netherlands	CENTER-TBI Investigator	
Bram	Jacobs			Department of Neurology, University Medical Center Groningen, Groningen, Netherlands	Groningen, Netherlands	CENTER-TBI Investigator	
Ewout W.	Steyerberg			Dept. of Department of Biomedical Data Sciences, Leiden University Medical Center, Leiden, The Netherlands	Leiden, Netherlands	CENTER-TBI Participant + MC	

Supplemental Online Content: Nonauthor Collaborators

*Indicates required information. Only first name, last name, and suffix will appear in PubMed.

*First Name and Middle Initial(s)	*Last Name	*Suffix (eg, Jr, III)	Academic Degrees	Institution	Location (city, state/province, country)	Role or Contribution, eg, chair, principal investigator	Group (if more than 1 Group listed in the byline) and/or Subgroup (eg, Steering Committee)
Ronald	Bartels			Department of Neurosurgery, Radboud University Medical Center, Nijmegen, The Netherlands	Nijmegen, Netherlands	CENTER-TBI Investigator	
Hugo	den Boogert			Department of Neurosurgery, Radboud University Medical Center, Nijmegen, The Netherlands	Nijmegen, Netherlands	CENTER-TBI Investigator	
Erwin	Kompanje			Department of Intensive Care and Department of Ethics and Philosophy of Medicine, Erasmus Medical Center, Rotterdam, The Netherlands	Rotterdam, Netherlands	CENTER-TBI Participant	
Marjolijn	Timmers			Department of Intensive Care and Department of Ethics and Philosophy of Medicine, Erasmus Medical Center, Rotterdam, The Netherlands	Rotterdam, Netherlands	CENTER-TBI Participant	
Kelly	Foks			Department of Neurology, Erasmus MC, Rotterdam, the Netherlands	Rotterdam, Netherlands	CENTER-TBI Participant + Investigator	
Iain	Haitsma			Department of Neurosurgery, Erasmus MC, Rotterdam, the Netherlands	Rotterdam, Netherlands	CENTER-TBI Investigator	
Victor	Volovici			Department of Neurosurgery, Erasmus MC, Rotterdam, the Netherlands	Rotterdam, Netherlands	CENTER-TBI Participant + Investigator	
Mathieu	van der Jagt			Department of Intensive Care Adults, Erasmus MC– University Medical Center Rotterdam, Rotterdam, the Netherlands	Rotterdam, Netherlands	CENTER-TBI Investigator	

Supplemental Online Content: Nonauthor Collaborators

*Indicates required information. Only first name, last name, and suffix will appear in PubMed.

***First Name and Middle Initial(s)**	***Last Name**	***Suffix (eg, Jr, III)**	Academic Degrees	Institution	Location (city, state/province, country)	Role or Contribution, eg, chair, principal investigator	Group (if more than 1 Group listed in the byline) and/or Subgroup (eg, Steering Committee)
Juanita A.	Haagsma			Department of Public Health, Erasmus Medical Center-University Medical Center, Rotterdam, The Netherlands	Rotterdam, Netherlands	CENTER-TBI Participant	
Ana	Mikolic			Department of Public Health, Erasmus Medical Center-University Medical Center, Rotterdam, The Netherlands	Rotterdam, Netherlands	CENTER-TBI Participant	
Hester	Lingsma			Department of Public Health, Erasmus Medical Center-University Medical Center, Rotterdam, The Netherlands	Rotterdam, Netherlands	CENTER-TBI Participant + MC	
Kimberley	Velt			Department of Public Health, Erasmus Medical Center-University Medical Center, Rotterdam, The Netherlands	Rotterdam, Netherlands	CENTER-TBI Participant	
Jilske	Huijben			Department of Public Health, Erasmus Medical Center-University Medical Center, Rotterdam, The Netherlands	Rotterdam, Netherlands	CENTER-TBI Participant	
Daphne	Voormolen			Department of Public Health, Erasmus Medical Center-University Medical Center, Rotterdam, The Netherlands	Rotterdam, Netherlands	CENTER-TBI Participant	
Daan	Nieboer			Department of Public Health, Erasmus Medical Center-University Medical Center, Rotterdam, The Netherlands	Rotterdam, Netherlands	CENTER-TBI Participant	

Supplemental Online Content: Nonauthor Collaborators

*Indicates required information. Only first name, last name, and suffix will appear in PubMed.

*First Name and Middle Initial(s)	*Last Name	*Suffix (eg, Jr, III)	Academic Degrees	Institution	Location (city, state/province, country)	Role or Contribution, eg, chair, principal investigator	Group (if more than 1 Group listed in the byline) and/or Subgroup (eg, Steering Committee)
Eveline	Wiegers			Department of Public Health, Erasmus Medical Center-University Medical Center, Rotterdam, The Netherlands	Rotterdam, Netherlands	CENTER-TBI Participant	
Ernest	van Veen			Department of Public Health, Erasmus Medical Center-University Medical Center, Rotterdam, The Netherlands	Rotterdam, Netherlands	CENTER-TBI Participant	
Dana	Pisica			Department of Public Health, Erasmus Medical Center-University Medical Center, Rotterdam, The Netherlands	Rotterdam, Netherlands	CENTER-TBI Participant	
Isabel	Retel Helmrich			Department of Public Health, Erasmus Medical Center-University Medical Center, Rotterdam, The Netherlands	Rotterdam, Netherlands	CENTER-TBI Participant	
Charlie	Sewalt			Department of Public Health, Erasmus Medical Center-University Medical Center, Rotterdam, The Netherlands	Rotterdam, Netherlands	CENTER-TBI Participant	
Benjamin	Gravesteijn			Department of Public Health, Erasmus Medical Center-University Medical Center, Rotterdam, The Netherlands	Rotterdam, Netherlands	CENTER-TBI Participant	
Suzanne	Polinder			Department of Public Health, Erasmus Medical Center-University Medical Center, Rotterdam, The Netherlands	Rotterdam, Netherlands	CENTER-TBI Participant	

Supplemental Online Content: Nonauthor Collaborators

*Indicates required information. Only first name, last name, and suffix will appear in PubMed.

***First Name and Middle Initial(s)**	***Last Name**	***Suffix (eg, Jr, III)**	Academic Degrees	Institution	Location (city, state/province, country)	Role or Contribution, eg, chair, principal investigator	Group (if more than 1 Group listed in the byline) and/or Subgroup (eg, Steering Committee)
Ewout W.	Steyerberg			Department of Public Health, Erasmus Medical Center-University Medical Center, Rotterdam, The Netherlands	Rotterdam, Netherlands	CENTER-TBI Participant + MC	
Dick	Tibboel			Intensive Care and Department of Pediatric Surgery, Erasmus Medical Center, Sophia Children's Hospital, Rotterdam, The Netherlands	Rotterdam, Netherlands	CENTER-TBI Participant	
Roel	van Wijk			Dept. of Neurosurgery, Leiden University Medical Center, Leiden, The Netherlands and Dept. of Neurosurgery, Medical Center Haaglanden, The Hague, The Netherlands	Leiden, Netherlands	CENTER-TBI Participant + Investigator	
Jeroen T.J.M.	van Dijck			Dept. of Neurosurgery, Leiden University Medical Center, Leiden, The Netherlands and Dept. of Neurosurgery, Medical Center Haaglanden, The Hague, The Netherlands	Leiden, Netherlands	CENTER-TBI Participant + Investigator	
Thomas A.	van Essen			Dept. of Neurosurgery, Leiden University Medical Center, Leiden, The Netherlands and Dept. of Neurosurgery, Medical Center Haaglanden, The Hague, The Netherlands	Leiden, Netherlands	CENTER-TBI Participant + Investigator	

*Indicates required information. Only first name, last name, and suffix will appear in PubMed.

*First Name and Middle Initial(s)	*Last Name	*Suffix (eg, Jr, III)	Academic Degrees	Institution	Location (city, state/province, country)	Role or Contribution, eg, chair, principal investigator	Group (if more than 1 Group listed in the byline) and/or Subgroup (eg, Steering Committee)
Wilco	Peul			Dept. of Neurosurgery, Leiden University Medical Center, Leiden, The Netherlands and Dept. of Neurosurgery, Medical Center Haaglanden, The Hague, The Netherlands	Leiden, Netherlands	CENTER-TBI Participant + Investigator + MC	
Guus	Schoonman			Department of Neurology, Elisabeth-TweeSteden Ziekenhuis, Tilburg, the Netherlands	Tilburg, Netherlands	CENTER-TBI Investigator	
Kelly	Jones			National Institute for Stroke and Applied Neurosciences, Faculty of Health and Environmental Studies, Auckland University of Technology, Auckland, New Zealand	Auckland, New Zealand	CENTER-TBI Participant	
Valery L.	Feigin			National Institute for Stroke and Applied Neurosciences, Faculty of Health and Environmental Studies, Auckland University of Technology, Auckland, New Zealand	Auckland, New Zealand	CENTER-TBI Participant	
Braden	Te Ao			National Institute for Stroke and Applied Neurosciences, Faculty of Health and Environmental Studies, Auckland University of Technology, Auckland, New Zealand	Auckland, New Zealand	CENTER-TBI Participant	

*Indicates required information. Only first name, last name, and suffix will appear in PubMed.

*First Name and Middle Initial(s)	*Last Name	*Suffix (eg, Jr, III)	Academic Degrees	Institution	Location (city, state/province, country)	Role or Contribution, eg, chair, principal investigator	Group (if more than 1 Group listed in the byline) and/or Subgroup (eg, Steering Committee)
Alice	Theadom			National Institute for Stroke and Applied Neurosciences, Faculty of Health and Environmental Studies, Auckland University of Technology, Auckland, New Zealand	Auckland, New Zealand	CENTER-TBI Participant	
Eirik	Helseth			Department of Neurosurgery, Oslo University Hospital, Oslo, Norway	Oslo, Norway	CENTER-TBI Participant + Investigator	
Cecilie	Roe			Department of Physical Medicine and Rehabilitation, Oslo University Hospital/University of Oslo, Oslo, Norway	Oslo, Norway	CENTER-TBI Participant + Investigator	
Olav	Roise			Division of Orthopedics, Oslo University Hospital, Oslo, Norway	Oslo, Norway	CENTER-TBI Participant + Investigator	
Olav	Roise			Institute of Clinical Medicine, Faculty of Medicine, Oslo University, Oslo, Norway	Oslo, Norway	CENTER-TBI Participant + Investigator	
Nada	Andelic			Division of Surgery and Clinical Neuroscience, Department of Physical Medicine and Rehabilitation, Oslo University Hospital and University of Oslo, Oslo, Norway	Oslo, Norway	CENTER-TBI Participant + Investigator	

Supplemental Online Content: Nonauthor Collaborators

*Indicates required information. Only first name, last name, and suffix will appear in PubMed.

*First Name and Middle Initial(s)	*Last Name	*Suffix (eg, Jr, III)	Academic Degrees	Institution	Location (city, state/province, country)	Role or Contribution, eg, chair, principal investigator	Group (if more than 1 Group listed in the byline) and/or Subgroup (eg, Steering Committee)
Shirin	Frisvold			Department of Anesthesiology and Intensive care, University Hospital Northern Norway, Tromso, Norway	Tromso, Norway	CENTER-TBI Investigator	
Lasse	Andreassen			Department of Neurosurgery, University Hospital Northern Norway, Tromso, Norway	Tromso, Norway	CENTER-TBI Participant + Investigator	
Audny	Anke			Department of Physical Medicine and Rehabilitation, University hospital Northern Norway, Tromso, Norway	Tromso, Norway	CENTER-TBI Participant + Investigator	
Anne	Vik			Department of Neuromedicine and Movement Science, Norwegian University of Science and Technology, NTNU, Trondheim, Norway	Trondheim, Norway	CENTER-TBI Investigator	
Toril	Skandsen			Department of Neuromedicine and Movement Science, Norwegian University of Science and Technology, NTNU, Trondheim, Norway	Trondheim, Norway	CENTER-TBI Investigator	
Anne	Vik			Department of Neurosurgery, St.Olavs Hospital, Trondheim University Hospital, Trondheim, Norway	Trondheim, Norway	CENTER-TBI Investigator	
Toril	Skandsen			Department of Physical Medicine and Rehabilitation, St.Olavs Hospital, Trondheim University Hospital, Trondheim, Norway	Trondheim, Norway	CENTER-TBI Investigator	

Supplemental Online Content: Nonauthor Collaborators

*Indicates required information. Only first name, last name, and suffix will appear in PubMed.

*First Name and Middle Initial(s)	*Last Name	*Suffix (eg, Jr, III)	Academic Degrees	Institution	Location (city, state/province, country)	Role or Contribution, eg, chair, principal investigator	Group (if more than 1 Group listed in the byline) and/or Subgroup (eg, Steering Committee)
Horia	Ples			Department of Neurosurgery, Emergency County Hospital Timisoara , Timisoara, Romania	Timisoara, Romania	CENTER-TBI Investigator	
Cristina Maria	Tudora			Department of Neurosurgery, Emergency County Hospital Timisoara , Timisoara, Romania	Timisoara, Romania	CENTER-TBI Investigator	
Ancuta	Negru			Department of Neurosurgery, Emergency County Hospital Timisoara , Timisoara, Romania	Timisoara, Romania	CENTER-TBI Investigator	
Peter	Vulekovic			Department of Neurosurgery, Clinical centre of Vojvodina, Faculty of Medicine, University of Novi Sad, Novi Sad, Serbia	Novi Sad, Serbia	CENTER-TBI Investigator	
Đula	Đilvesi			Department of Neurosurgery, Clinical centre of Vojvodina, Faculty of Medicine, University of Novi Sad, Novi Sad, Serbia	Novi Sad, Serbia	CENTER-TBI Investigator	
Mladen	Karan			Department of Neurosurgery, Clinical centre of Vojvodina, Faculty of Medicine, University of Novi Sad, Novi Sad, Serbia	Novi Sad, Serbia	CENTER-TBI Investigator	
Jagoš	Golubović			Department of Neurosurgery, Clinical centre of Vojvodina, Faculty of Medicine, University of Novi Sad, Novi Sad, Serbia	Novi Sad, Serbia	CENTER-TBI Investigator	
Veronika	Rehorčíková			Department of Public Health, Faculty of Health Sciences and Social Work, Trnava University, Trnava, Slovakia	Trnava, Slovak Republic	CENTER-TBI Participant	

*Indicates required information. Only first name, last name, and suffix will appear in PubMed.

***First Name and Middle Initial(s)**	***Last Name**	***Suffix (eg, Jr, III)**	Academic Degrees	Institution	Location (city, state/province, country)	Role or Contribution, eg, chair, principal investigator	Group (if more than 1 Group listed in the byline) and/or Subgroup (eg, Steering Committee)
Mark Steven	Taylor			Department of Public Health, Faculty of Health Sciences and Social Work, Trnava University, Trnava, Slovakia	Trnava, Slovak Republic	CENTER-TBI Participant	
Alexandra	Brazinova			Department of Public Health, Faculty of Health Sciences and Social Work, Trnava University, Trnava, Slovakia	Trnava, Slovakia	CENTER-TBI Participant	
Marek	Majdan			Department of Public Health, Faculty of Health Sciences and Social Work, Trnava University, Trnava, Slovakia	Trnava, Slovakia	CENTER-TBI Participant	
Juan	Sahuquillo			Department of Neurosurgery, Vall d'Hebron University Hospital, Barcelona, Spain	Barcelona, Spain	CENTER-TBI Investigator	
Andreea	Rădoi			Neurotraumatology and Neurosurgery Research Unit (UNINN), Vall d'Hebron Research Institute, Barcelona, Spain	Barcelona, Spain	CENTER-TBI Investigator	
Guillermo	Carbayo Lozano			Department of Neurosurgery, Hospital of Cruces, Bilbao, Spain	Bilbao, Spain	CENTER-TBI Investigator	
Inigo	Pomposo			Department of Neurosurgery, Hospital of Cruces, Bilbao, Spain	Bilbao, Spain	CENTER-TBI Investigator	
Alfonso	Lagares			Department of Neurosurgery, Hospital Universitario 12 de Octubre, Madrid, Spain	Madrid, Spain	CENTER-TBI Investigator	

*Indicates required information. Only first name, last name, and suffix will appear in PubMed.

***First Name and Middle Initial(s)**	***Last Name**	***Suffix (eg, Jr, III)**	Academic Degrees	Institution	Location (city, state/province, country)	Role or Contribution, eg, chair, principal investigator	Group (if more than 1 Group listed in the byline) and/or Subgroup (eg, Steering Committee)
Pedro A.	Gomez			Department of Neurosurgery, Hospital Universitario 12 de Octubre, Madrid, Spain	Madrid, Spain	CENTER-TBI Investigator	
Ana M.	Castaño-León			Department of Neurosurgery, Hospital Universitario 12 de Octubre, Madrid, Spain	Madrid, Spain	CENTER-TBI Investigator	
Pablo	Gagliardo			Fundación Instituto Valenciano de Neurorrehabilitación (FIVAN), Valencia, Spain	Valencia, Spain	CENTER-TBI Associated Participant	
Matej	Oresic			School of Medical Sciences, Örebro University, Örebro, Sweden	Örebro, Sweden	CENTER-TBI Participant	
Bo-Michael	Bellander			Deparment of Neurosurgery & Anesthesia & intensive care medicine, Karolinska University Hospital, Stockholm, Sweden	Stockholm, Sweden	CENTER-TBI Investigator	
Linda	Lanyon			Karolinska Institutet, INCF International Neuroinformatics Coordinating Facility, Stockholm, Sweden	Stockholm, Sweden	CENTER-TBI Participant + MC	
Pradeep	George			Karolinska Institutet, INCF International Neuroinformatics Coordinating Facility, Stockholm, Sweden	Stockholm, Sweden	CENTER-TBI Participant	
Visakh	Muraleedharan			Karolinska Institutet, INCF International Neuroinformatics Coordinating Facility, Stockholm, Sweden	Stockholm, Sweden	CENTER-TBI Participant + DCTF	

*Indicates required information. Only first name, last name, and suffix will appear in PubMed.

***First Name and Middle Initial(s)**	***Last Name**	***Suffix (eg, Jr, III)**	Academic Degrees	Institution	Location (city, state/province, country)	Role or Contribution, eg, chair, principal investigator	Group (if more than 1 Group listed in the byline) and/or Subgroup (eg, Steering Committee)
David	Nelson			Karolinska Institutet, INCF International Neuroinformatics Coordinating Facility, Stockholm, Sweden	Stockholm, Sweden	CENTER-TBI Participant + Investigator	
Cecilia	Ackerlund			Karolinska Institutet, INCF International Neuroinformatics Coordinating Facility, Stockholm, Sweden	Stockholm, Sweden	CENTER-TBI Participant + Investigator	
Lars-Owe	Koskinen			Department of Neurosurgery, Umea University, Umea, Sweden	Umea, Sweden	CENTER-TBI Investigator	
Nina	Sundström			Department of Radiation Sciences, Biomedical Engineering, Umea University, Umea, Sweden	Umea, Sweden	CENTER-TBI Investigator	
Camilla	Brorsson			Department of Surgery and Perioperative Science, Umea University, Umea, Sweden	Umea, Sweden	CENTER-TBI Investigator	
Antonio	Belli			NIHR Surgical Reconstruction and Microbiology Research Centre, Birmingham, UK	Birmingham, UK	CENTER-TBI Investigator	
Alex	Manara			Intensive Care Unit, Southmead Hospital, Bristol, Bristol, UK	Bristol, UK	CENTER-TBI Investigator	
Matt	Thomas			Intensive Care Unit, Southmead Hospital, Bristol, Bristol, UK	Bristol, UK	CENTER-TBI Investigator	
Marek	Czosnyka			Brain Physics Lab, Division of Neurosurgery, Dept of Clinical Neurosciences, University of Cambridge, Addenbrooke’s Hospital, Cambridge, UK	Cambridge, UK	CENTER-TBI Participant + Investigator	

Supplemental Online Content: Nonauthor Collaborators

*Indicates required information. Only first name, last name, and suffix will appear in PubMed.

*First Name and Middle Initial(s)	*Last Name	*Suffix (eg, Jr, III)	Academic Degrees	Institution	Location (city, state/province, country)	Role or Contribution, eg, chair, principal investigator	Group (if more than 1 Group listed in the byline) and/or Subgroup (eg, Steering Committee)
Peter	Smielewski			Brain Physics Lab, Division of Neurosurgery, Dept of Clinical Neurosciences, University of Cambridge, Addenbrooke's Hospital, Cambridge, UK	Cambridge, UK	CENTER-TBI Participant + Investigator	
Manuel	Cabeleira			Brain Physics Lab, Division of Neurosurgery, Dept of Clinical Neurosciences, University of Cambridge, Addenbrooke's Hospital, Cambridge, UK	Cambridge, UK	CENTER-TBI Participant + Investigator	
Jonathan	Coles			Department of Anesthesia & Neurointensive Care, Cambridge Universiyt Hospital NHS Foundation Trust, Cambridge, UK	Cambridge, UK	CENTER-TBI Participant + Investigator	
Sylvia	Richardson			Director, MRC Biostatistics Unit, Cambridge Institute of Public Health, Cambridge, UK	Cambridge, UK	CENTER-TBI Participant	
Frederick A.	Zeiler			Division of Anaesthesia, University of Cambridge, Addenbrooke's Hospital, Cambridge, UK	Cambridge, UK	CENTER-TBI Participant	
Emmanuel	Stamatakis			Division of Anaesthesia, University of Cambridge, Addenbrooke's Hospital, Cambridge, UK	Cambridge, UK	CENTER-TBI Participant	
Guy	Williams			Division of Anaesthesia, University of Cambridge, Addenbrooke's Hospital, Cambridge, UK	Cambridge, UK	CENTER-TBI Participant	

*Indicates required information. Only first name, last name, and suffix will appear in PubMed.

***First Name and Middle Initial(s)**	***Last Name**	***Suffix (eg, Jr, III)**	Academic Degrees	Institution	Location (city, state/province, country)	Role or Contribution, eg, chair, principal investigator	Group (if more than 1 Group listed in the byline) and/or Subgroup (eg, Steering Committee)
David	Menon			Division of Anaesthesia, University of Cambridge, Addenbrooke's Hospital, Cambridge, UK	Cambridge, UK	CENTER-TBI Participant + Investigator + MC	
Ari	Ercole			Division of Anaesthesia, University of Cambridge, Addenbrooke's Hospital, Cambridge, UK	Cambridge, UK	CENTER-TBI Participant + Investigator	
Abhishek	Dixit			Division of Anaesthesia, University of Cambridge, Addenbrooke's Hospital, Cambridge, UK	Cambridge, UK	CENTER-TBI Participant + Investigator	
Virginia	Newcombe			Division of Anaesthesia, University of Cambridge, Addenbrooke's Hospital, Cambridge, UK	Cambridge, UK	CENTER-TBI Participant + Investigator	
Sophie	Richter			Division of Anaesthesia, University of Cambridge, Addenbrooke's Hospital, Cambridge, UK	Cambridge, UK	CENTER-TBI Participant + Investigator	
Charles	McFadyen			Division of Anaesthesia, University of Cambridge, Addenbrooke's Hospital, Cambridge, UK	Cambridge, UK	CENTER-TBI Participant + Investigator	
Peter J.	Hutchinson			Division of Neurosurgery, Department of Clinical Neurosciences, Addenbrooke's Hospital & University of Cambridge, Cambridge, UK	Cambridge, UK	CENTER-TBI Participant + Investigator	

Supplemental Online Content: Nonauthor Collaborators

*Indicates required information. Only first name, last name, and suffix will appear in PubMed.

***First Name and Middle Initial(s)**	***Last Name**	***Suffix (eg, Jr, III)**	Academic Degrees	Institution	Location (city, state/province, country)	Role or Contribution, eg, chair, principal investigator	Group (if more than 1 Group listed in the byline) and/or Subgroup (eg, Steering Committee)
Angelos G.	Kolias			Division of Neurosurgery, Department of Clinical Neurosciences, Addenbrooke's Hospital & University of Cambridge, Cambridge, UK	Cambridge, UK	CENTER-TBI Participant + Investigator	
Hadie	Adams			Division of Neurosurgery, Department of Clinical Neurosciences, Addenbrooke's Hospital & University of Cambridge, Cambridge, UK	Cambridge, UK	CENTER-TBI Participant + Investigator	
Marta	Correia			Radiology/MRI department, MRC Cognition and Brain Sciences Unit, Cambridge, UK	Cambridge, UK	CENTER-TBI Associated Participant	
Jonathan	Rhodes			Department of Anaesthesia, Critical Care & Pain MedicineNHS Lothian & University of Edinburg, Edinburgh, UK	Edinburgh, UK	CENTER-TBI Investigator	
William	Stewart			Department of Neuropathology, Queen Elizabeth University Hospital and University of Glasgow , Glasgow, UK	Glasgow, UK	CENTER-TBI Participant	
Catherine	McMahon			Department of Neurosurgery, The Walton centre NHS Foundation Trust, Liverpool, UK	Liverpool, UK	CENTER-TBI Investigator	
Daniel	Rueckert			Department of Computing, Imperial College London, London, UK	London, UK	CENTER-TBI Participant	

*Indicates required information. Only first name, last name, and suffix will appear in PubMed.

*First Name and Middle Initial(s)	*Last Name	*Suffix (eg, Jr, III)	Academic Degrees	Institution	Location (city, state/province, country)	Role or Contribution, eg, chair, principal investigator	Group (if more than 1 Group listed in the byline) and/or Subgroup (eg, Steering Committee)
Ben	Glocker			Department of Computing, Imperial College London, London, UK	London, UK	CENTER-TBI Participant	
Christos	Tolias			Department of Neurosurgery, Kings college London, London, UK	London, UK	CENTER-TBI Investigator	
Helen	Dawes			Movement Science Group, Faculty of Health and Life Sciences, Oxford Brookes University, Oxford, UK	Oxford, UK	CENTER-TBI Participant	
Patrick	Esser			Movement Science Group, Faculty of Health and Life Sciences, Oxford Brookes University, Oxford, UK	Oxford, UK	CENTER-TBI Participant	
Caroline	van Heugten			Movement Science Group, Faculty of Health and Life Sciences, Oxford Brookes University, Oxford, UK	Oxford, UK	CENTER-TBI Participant	
Nicola	Curry			Oxford University Hospitals NHS Trust, Oxford, UK	Oxford, UK	CENTER-TBI Participant	
Simon	Stanworth			Oxford University Hospitals NHS Trust, Oxford, UK	Oxford, UK	CENTER-TBI Participant	
Fiona	Lecky			Centre for Urgent and Emergency Care Research (CURE), Health Services Research Section, School of Health and Related Research (ScHARR), University of Sheffield, Sheffield, UK	Sheffield, UK	CENTER-TBI Participant + Investigator + MC	

*Indicates required information. Only first name, last name, and suffix will appear in PubMed.

*First Name and Middle Initial(s)	*Last Name	*Suffix (eg, Jr, III)	Academic Degrees	Institution	Location (city, state/province, country)	Role or Contribution, eg, chair, principal investigator	Group (if more than 1 Group listed in the byline) and/or Subgroup (eg, Steering Committee)
Fiona	Lecky			Emergency Department, Salford Royal Hospital, Salford UK	Salford, UK	CENTER-TBI Participant + Investigator + MC	
Olubukola	Otesile			Centre for Urgent and Emergency Care Research (CURE), Health Services Research Section, School of Health and Related Research (ScHARR), University of Sheffield, Sheffield, UK	Sheffield, UK	CENTER-TBI Participant + Investigator	
Faye	Johnson			Salford Royal Hospital NHS Foundation Trust Acute Research Delivery Team, Salford, UK	Salford, UK	CENTER-TBI Investigator	
Paul	Dark			University of Manchester NIHR Biomedical Research Centre, Critical Care Directorate, Salford Royal Hospital NHS Foundation Trust, Salford, UK	Salford, UK	CENTER-TBI Investigator	
Stefan	Jankowski			Neurointensive Care , Sheffield Teaching Hospitals NHS Foundation Trust, Sheffield, UK	Sheffield, UK	CENTER-TBI Investigator	
Roger	Lightfoot			Department of Anesthesiology & Intensive Care, University Hospitals Southhampton NHS Trust, Southhampton, UK	Southhampton, UK	CENTER-TBI Investigator	
Lindsay	Wilson			Division of Psychology, University of Stirling, Stirling, UK	Stirling, UK	CENTER-TBI Participant	

Supplemental Online Content: Nonauthor Collaborators

*Indicates required information. Only first name, last name, and suffix will appear in PubMed.

*First Name and Middle Initial(s)	*Last Name	*Suffix (eg, Jr, III)	Academic Degrees	Institution	Location (city, state/province, country)	Role or Contribution, eg, chair, principal investigator	Group (if more than 1 Group listed in the byline) and/or Subgroup (eg, Steering Committee)
Lindsay	Horton			Division of Psychology, University of Stirling, Stirling, UK	Stirling, UK	CENTER-TBI Participant	
Robert	Stevens			Division of Neuroscience Critical Care, John Hopkins University School of Medicine, Baltimore, USA	Baltimore, USA	CENTER-TBI Associated Participant	
Aarno	Palotie			Analytic and Translational Genetics Unit, Department of Medicine; Psychiatric & Neurodevelopmental Genetics Unit, Department of Psychiatry; Department of Neurology, Massachusetts General Hospital, Boston, MA, USA	Boston MA, USA	CENTER-TBI Participant	
Jonathan	Rosand			Broad Institute, Cambridge MA Harvard Medical School, Boston MA, Massachusetts General Hospital, Boston MA, USA	Boston MA, USA	CENTER-TBI Associated Participant	
Geoffrey	Manley			Department of Neurological Surgery, University of California, San Francisco, California, USA	California, USA	CENTER-TBI Participant	
Mike	Jarrett			Quesgen Systems Inc., Burlingame, California, USA	California, USA	CENTER-TBI Participant	
Vibeke	Brinck			Quesgen Systems Inc., Burlingame, California, USA	California, USA	CENTER-TBI Participant	

Supplemental Online Content: Nonauthor Collaborators

*Indicates required information. Only first name, last name, and suffix will appear in PubMed.

*First Name and Middle Initial(s)	*Last Name	*Suffix (eg, Jr, III)	Academic Degrees	Institution	Location (city, state/province, country)	Role or Contribution, eg, chair, principal investigator	Group (if more than 1 Group listed in the byline) and/or Subgroup (eg, Steering Committee)
Aarno	Palotie			Program in Medical and Population Genetics; The Stanley Center for Psychiatric Research, The Broad Institute of MIT and Harvard, Cambridge, MA, USA	Cambridge MA, USA	CENTER-TBI Participant	
Kevin K.W.	Wang			Department of Emergency Medicine, University of Florida, Gainesville, Florida, USA	Florida, USA	CENTER-TBI Participant	
Zhihui	Yang			Department of Emergency Medicine, University of Florida, Gainesville, Florida, USA	Florida, USA	CENTER-TBI Participant	
Paul M.	Vespa			Director of Neurocritical Care, University of California, Los Angeles, USA	Los Angeles, USA	CENTER-TBI Associated Participant	
